# Organic contaminants and atmospheric nitrogen at the graphene–water interface: a simulation study†

**DOI:** 10.1039/d1na00570g

**Published:** 2022-03-16

**Authors:** Ravindra Thakkar, Sandun Gajaweera, Jeffrey Comer

**Affiliations:** Nanotechnology Innovation Center of Kansas State, Department of Anatomy and Physiology 1620 Denison Avenue Mahattan Kansas USA jeffcomer@ksu.edu

## Abstract

Ordered nanoscale patterns have been observed by atomic force microscopy at graphene–water and graphite–water interfaces. The two dominant explanations for these patterns are that (i) they consist of self-assembled organic contaminants or (ii) they are dense layers formed from atmospheric gases (especially nitrogen). Here we apply molecular dynamics simulations to study the behavior of dinitrogen and possible organic contaminants at the graphene–water interface. Despite the high concentration of N_2_ in ambient air, we find that its expected occupancy at the graphene–water interface is quite low. Although dense (disordered) aggregates of dinitrogen have been observed in previous simulations, our results suggest that they are stable only in the presence of supersaturated aqueous N_2_ solutions and dissipate rapidly when they coexist with nitrogen gas near atmospheric pressure. On the other hand, although heavy alkanes are present at only trace concentrations (micrograms per cubic meter) in typical indoor air, we predict that such concentrations can be sufficient to form ordered monolayers that cover the graphene–water interface. For octadecane, grand canonical Monte Carlo suggests nucleation and growth of monolayers above an ambient concentration near 6 μg m^−3^, which is less than some literature values for indoor air. The thermodynamics of the formation of these alkane monolayers includes contributions from the hydration free-energy (unfavorable), the free-energy of adsorption to the graphene–water interface (highly favorable), and integration into the alkane monolayer phase (highly favorable). Furthermore, the peak-to-peak distances in AFM force profiles perpendicular to the interface (0.43–0.53 nm), agree with the distances calculated in simulations for overlayers of alkane-like molecules, but not for molecules such as N_2_, water, or aromatics. Taken together, these results suggest that ordered domains observed on graphene, graphite, and other hydrophobic materials in water are consistent with alkane-like molecules occupying the interface.

## Abstract 2

Formation of hydrocarbon monolayers at the graphene–water interface can be predicted from the concentration in ambient air and the free energies of hydration, adsorption from the aqueous phase, and subsequent integration into the monolayer phase.

## Graphical abstract

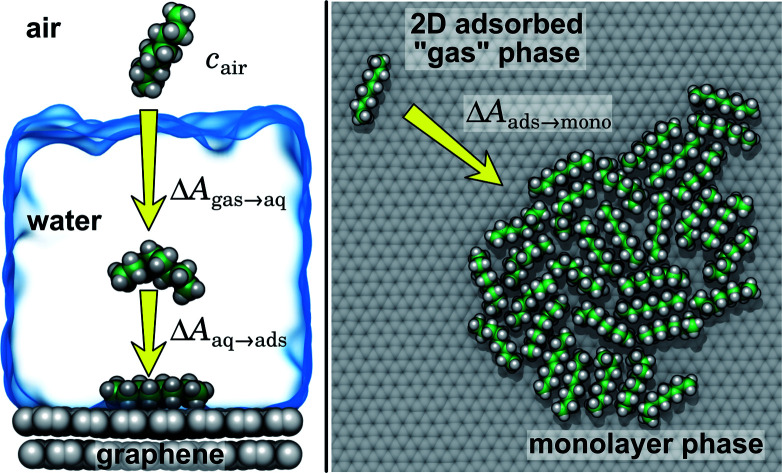

## Introduction

The behavior of graphitic surfaces in various media is important for technological applications of graphite, graphene, and carbon nanotubes. However, despite several decades of study and the topographical and chemical simplicity of the graphene and graphene-like surfaces, the detailed structure of graphene–water and graphite–water interfaces under typical experimental conditions remains controversial. In particular, contact angle^1,2^ and capacitance measurements,^3^ infrared spectroscopy,^2^ and atomic force microscopy (AFM)^4–11^ suggest the presence of contaminants of some nature that rapidly accumulate on even freshly cleaved graphite surfaces exposed to water or air under typical laboratory conditions. The identity of these contaminants remains somewhat unclear and likely depends on the details of the environment to which the sample has been exposed; however, two major hypotheses are that these contaminants consist of (i) a mixture of hydrocarbon species or (ii) a condensed form of a major atmospheric gas, with most attention being given to N_2_. These two hypothesized compositions are not necessarily mutually exclusive, although one of the two or neither might be the dominant component.

### The ubiquity of organic contaminants

Volatile organic compounds (VOCs), of both natural and artificial origin, are present at low concentrations in indoor^12,13^ and outdoor air,^14^ and emanate from polymeric materials^15^ and human breath.^16^ These VOCs have many potential sources in the laboratory environment including air, apparatus materials, and the researchers themselves; hence, preventing contamination by these compounds is especially difficult.^17^ The most prominent VOCs are hydrocarbons and their simple derivatives with alcohol, aldehyde, ketone, ester, and chloro groups, with typical per-species concentrations <50 μg m^−3^.^12,13,17^ Many of these VOCs have a high affinity for graphitic surfaces and the graphitic–water interface^18–20^ and can be expected to reach appreciable concentrations at these interfaces despite the low ambient chemical potential.

As early as 1975, hydrocarbon contamination from ambient air was posited to explain discrepancies in measurements of the contact angle of water droplets on graphite, with larger angles attributed to the accumulation of hydrophobic molecules on the graphite surface.^1^ More recently, Kozbial *et al.*^2^ reported that the water contact angle on freshly cleaved graphite and freshly synthesized graphene increased from ≈60° to ≈90° after a few minutes of exposure to air. This increase in contact angle was associated with the appearance of methylene stretching peaks in the infrared spectrum, indicating the presence of adsorbed molecules similar to linear alkanes.

When graphitic carbon is studied in an aqueous environment, VOCs either originally present in the aqueous solution or migrating into the solution from air may adsorb to the graphite–water interface. Hurst *et al.*^3^ detected decreases in the capacitance of freshly cleaved graphite samples in water in as little as 10 minutes, which was attributed to adsorption of hydrocarbon contaminants. On the other hand, low temperature storage and high humidity was shown to slow the accumulation of hydrophobic species on graphite.^21^

### Atomic force microscopy at aqueous hydrophobic interfaces

Many groups have reported AFM images including curious striped domains on graphitic surfaces and at graphene–water and graphite–water interfaces.^8^ The geometry of these domains appears to vary, with stripe widths ranging from 2 to 5 nm. Consistent with the ubiquity of hydrocarbon contaminants described above, these domains have been proposed to be layers of hydrocarbons.^4,6–8^ This hypothesis is supported by the work Seibert *et al.*,^8^ which showed formation of such domains when plastic syringes were used, but not when glass syringes were used, suggesting that the domains are composed of organic molecules either native to the plastic or adsorbed to the plastic from ambient air. Similarly, Berkelaar *et al.*^22^ argued that some objects identified as gaseous bubbles in AFM images might be due to polydimethylsiloxane (PDMS) used in syringes.

In contrast, other researchers have attributed the stripe domains to ordered arrangements of condensed N_2_ (or possibly O_2_) molecules.^5,9–11,23,24^ A role for N_2_ was supported by the fact that the domains grew rapidly when N_2_ gas was passed over the graphite, but much more slowly when O_2_ or Ar gases were used.^9^ Schlesinger and Sivan^24^ argued that organic contaminants can be ruled out as the constituents of the striped domains since degassing caused the layers to disappear and measurements of the total carbon concentration showed insufficient carbon to form the layers. Furthermore, simulations have shown significant enhancement of N_2_ concentration at the graphene–water interface from its concentration in bulk water.^10,25^

Recent work by the Garcia group using 3D AFM^6,26^ provides key information about the structure and possible identity of these layers. As a function of distance from the solid–liquid interface, AFM measurements show oscillatory behavior of the measured force, which becomes less pronounced at greater distances. The force profiles in water on relatively hydrophobic materials (graphene, MoS_2_, and WSe_2_) showed more pronounced undulations with larger wavelengths (0.43–0.53 nm) than those above hydrophilic mica (0.33–0.34 nm).^6^ This suggests that molecules larger than water might be present at the graphene surface even when the graphene is nominally immersed in pure water. Strikingly, shorter wavelengths, similar to those on mica, were sometimes measured when freshly cleaved graphite was immersed in pure water within two seconds of cleavage.^26^ The values found for graphite 30 minutes after cleavage were more consistent and settled on larger values (≈0.5 nm) suggesting it took some time for the molecules conjectured to occupy the interface to collect there. Furthermore, the typical striped pattern parallel to the interface was found to be associated with this time-dependent interfacial layer. On the other hand, no such time-dependent differences were observed in the force profiles for fresh and aged mica. Another notable result of this work is that the force profile for graphite immersed in nominally pure water was nearly identical to that immersed in hexane, suggesting that the molecules occupying the interface might be somehow similar to hexane.

### Molecular simulations of the graphene–water interface

Our molecular dynamics simulations showed that the force on a model AFM tip has the same wavelength as undulations in solvent density and that the undulations in solvent density are characteristic of particular classes of molecules. Therefore, the AFM profiles in the direction perpendicular to the surface might yield information on the chemical nature of the unknown molecules. We calculated the mean force on an atomic model of an AFM tip asperity and observed excellent agreement between the calculated and measured force profiles for mica–water and graphite–hexane systems.^26^ As might be expected from the discussion in the preceding paragraph, a naïve model of the graphite–water interface, including only graphene sheets and water, did not yield a force profile in good agreement with the AFM experiments in nominally pure water. On the other hand, the calculated force profile for the graphite–hexane system agreed well with the nominally graphite–water experiments. Again, this suggested that the graphite–water interface was occupied by some contaminant.

We also found that the wavelength of force undulations calculated using the AFM tip asperity model agreed well with undulations of the mass density of solvent layers at the interface. For example, straight-chain alkanes, regardless of molecular mass, exhibited density undulations with a consistent wavelength of 0.45 nm.^26^ Hence, we hypothesized that the molecules occupying the graphite–water interface in the experiments consisted mostly of alkane moieties although small amounts of other chemical groups may be present. On the other hand, water and N_2_ exhibit density undulations on a significantly shorter scale and therefore are unlikely to be the predominant components of the interfacial layer.

Other groups have simulated N_2_ at the graphene–water interface to explore the N_2_ hypothesis. Peng and *et al.*^25^ investigated N_2_ adsorption at this interface using molecular dynamics simulations and reported aggregates of N_2_ and dense gas layers with densities several orders of magnitude greater than that in air. The simulations of Wang *et al.* showed similar results.^10^ Below we argue that these simulations represent supersaturated N_2_–water solutions, and we demonstrate that the dense N_2_ phase rapidly dissipates with a different simulation approach.

Despite many simulation studies of adsorption of particular organic molecules at interfaces between water and graphitic surfaces,^19,26–31^ to our knowledge, molecular dynamics simulations have not yet been applied to study possible hydrocarbon contaminants at the graphene–water interface or the behavior of mixtures of N_2_ and hydrocarbons at this interface. While large organic molecules can be expected to have a high affinity for the graphite–water and graphene–water interfaces, they are present at only trace concentrations in ambient air. On the other hand, the ambient concentrations of atmospheric gases such as N_2_, O_2_, and Ar are many orders of magnitude higher, but their interaction with the interface is much weaker. Hence, it is difficult to determine by qualitative arguments whether organic molecules or atmospheric gases such as N_2_ might dominate at the graphite–water interface. The goal of the present study is to leverage molecular dynamics simulations and free energy calculations to provide semiquantitative estimates of the propensity of ambient VOCs and N_2_ to occupy the graphene–water interface and compare the structural properties predicted in simulations to observational data.

It should be noted that, in the context of physical adsorption of neutral organic molecules at room temperature, surfaces of graphene, the graphite basal plane (0001), and even large-diameter carbon nanotubes behave quite similarly. The adsorption characteristics of other graphite planes, such as (1010), are expected to be quite distinct. We explored the effects of the number of graphene layers and the curvature of the surface (as in carbon nanotubes) in previous work.^19,20^ We found a small (<*k*_B_*T*/2 in magnitude) but measurable effect on adsorption free energy between the basal plane of 4-layer graphite and a single graphene sheet surrounded by water on both sides.^19^ Here, all simulations were performed with a flat graphene bilayer (except those including a defect-rich graphene sheet).

## Results and discussion

### N_2_ binds only weakly to the graphene–water interface

**Fig. 1:**
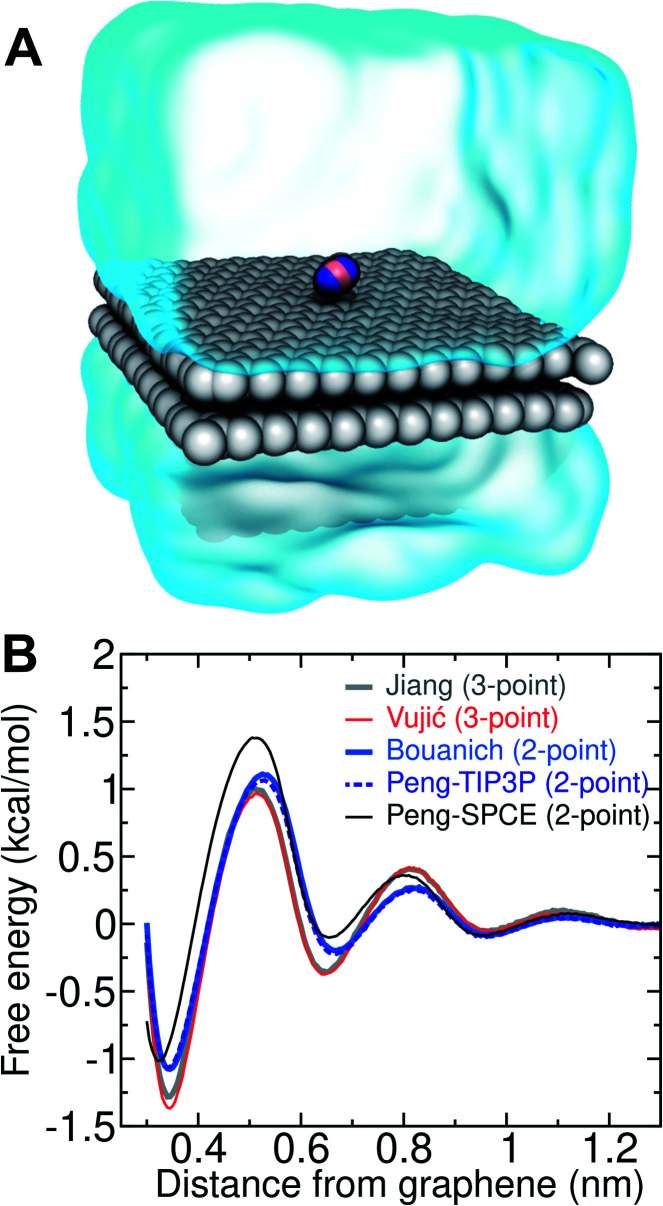
Free-energy calculations for N_2_ at the graphene–water interface. (A) Snapshot of an exemplary simulation system. Graphene carbon is shown as gray spheres, and nitrogen atoms are shown as blue spheres. This system includes a 3-point N_2_ model, where a massless positive point charge (pink sphere) lies at the centroid of the nitrogen atoms. Although the simulations included explicit water molecules, for clarity, they are represented here as a translucent surface. (B) Free energy as a function of distance from the upper graphene layer for five different models of N_2_, water, and graphene.

Several different classical models of dinitrogen have been developed, including chargeless 2-point models^25,32^ and models consisting of 3 point charges that reproduce the molecular quadrupole moment of N_2_.^33,34^ To our knowledge, there has been no optimization of any N_2_ models in the context of interactions at the graphene–water interface. Hence, we performed free energy calculations to compare the behavior of different N_2_ models. We included the 3-point model developed by Jiang and Sandler^33^ (Jiang), a reoptimization of the latter model by Vujić and Lyubartsev^34^ (Vujić), the 2-point model developed Bouanich^32^ (Bouanich), the 2-point model used by Peng *et al.*^25^ (Peng-TIP3P) and another set of parameters using the same dinitrogen, water, and graphitic carbon model as these authors (Peng-SPCE), allowing for direct comparison to their results. Except for this latter case, we used the CHARMM TIP3P water model and CHARMM^35^ parameters for graphitic carbon. The potentials of mean force as a function of the distance between the N_2_ molecule and upper graphene layer are shown in Fig. 1. In all cases, the free energy at the interface is associated with only weak binding, with a minimum free energy for transfer from the aqueous phase of Δ*A*_aq→ads_ ≥ −1.4 kcal mol^−1^.

The calculations described in Fig. 1 are performed with a single N_2_ molecule; hence, interactions between N_2_ molecules are not considered. In the limit of negligible adsorbate–adsorbate interactions we can estimate the areal number density of N_2_ molecules at the graphene–water interface from Fig. 1B, which is calculated by1
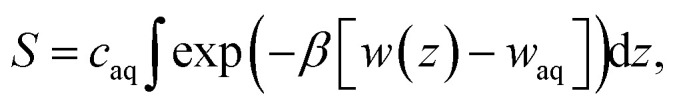
where *c*_aq_ = 3.08 × 10^−4^ molecules per nm^3^ (or 0.512 mmol L^−1^) is the number density of N_2_ in pure water at room temperature in contact with the atmosphere,^36,37^*β* = 1/(*k*_B_*T*) is the inverse thermal energy, *w*(*z*) is a potential of mean force like those plotted in Fig. 1B, and *w*_aq_ is the value of *w*(*z*) far from the interface, where the water becomes bulk-like and isotropic (*w*_aq_ = 0 by the convention used in Fig. 1B). The integral runs from the graphene surface (*z*_graph_) to a distance above the surface where the water is bulk-like (*z*_water_). Depending on the computational model, applying eqn (1) gives an N_2_ density ranging from 380 to 510 molecules per μm^2^, which is clearly insufficient to explain the appearance of ordered layers of molecules in AFM. Any clustering of N_2_ molecules would have be due to cooperative interactions or nonequilibrium processes, which we explore further below.

### N_2_ aggregates dissipate in the absence of supersaturation

A number of authors have proposed that the stripes observed by AFM are high-density ordered gas layers that spontaneously form at the graphene–water interface.^9,11^ In simulations, Peng *et al.*^25^ and Wang *et al.*^10^ have reported “dense gas layers” with concentrations of N_2_ many times above atmospheric concentrations at the graphene–water interface, although these layers appear disordered. Similar to Peng *et al.*, we found that hemispherical aggregates (Fig. S1 of the ESI†) of N_2_ spontaneously form at the graphene–water interface at relatively low areal densities of N_2_, using the same N_2_, water, and graphene models as these authors. Within the aggregate, the density is a nearly uniform 7.1 molecules per nm^3^, which is ≈370 times the number density of N_2_ in the atmosphere ([N_2_(g)]_atmos_ = 0.0194 nm^−3^). We also find that the stability of the N_2_ aggregates varies considerably with the model used for N_2_, water, and graphene. While the Peng-SPCE model exhibits dissolution of a hemispherical aggregate on the 100 ns time scale, the same aggregate dissipates within 15 ns using the Vujić model (Fig. S2 of the ESI†).

During the simulation illustrated in Fig. S1 of the ESI,† the water became supersaturated with dissolved N_2_ and the concentration plateaued at about 150 mmol L^−1^. This concentration is many times the equilibrium concentration water in contact with the atmosphere derived from experiment^36,37^ ([N_2_(aq)]_atmos_ = 0.512 mmol L^−1^) or simulations (0.22 mmol L^−1^, calculated from Δ*A*_gas→aq_ in Table 1). Previous simulations by other authors showing apparently stable N_2_ aggregates have also included supersaturated aqueous N_2_. For the system size considered in Fig. S1,† as well as those considered by Peng *et al.*^25^ and Wang *et al.*,^10^ fewer than one N_2_ molecule should be present on average in the aqueous phase at equilibrium on under standard conditions. However, Fig. S1† and figures in these other publications show many aqueous N_2_ molecules. The reason for this supersaturation is that it is difficult for aqueous N_2_ molecules to coalesce and form a gas phase on the simulation time scale, even when a barostatting algorithm is used to keep the system at atmospheric pressure. Supersaturation of N_2_ may also be relevant in experiments as well.

**Table 1:** Thermodynamics of adsorption to the graphene–water interface by N_2_ and selected VOCs. The table includes hydration free energies derived from the literature 
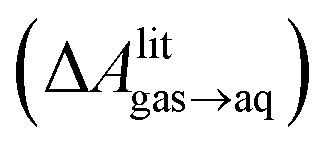
 and this work 
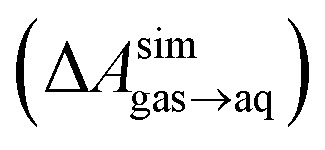
, free energies of adsorption at the graphene–water interface (Δ*A*_aq→ads_ and *λ*_ads_), free energies for transfer of an adsorbed molecule from an isolated phase to a dense monolayer phase (Δ*A*_ads→mono_), and the critical ambient concentration at which the graphene–water interface becomes completely covered with a monolayer (*c*_monolayer_). 
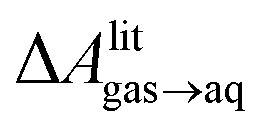
 is calculated from Henry's law constants given by Sander *et al.*,^37^ using either experimental^36,42^ or QSPR predicted values.^43^ The experimental values vary among sources;^37^ we chose what appeared to consensus values. For C10–C16 alkanes, 
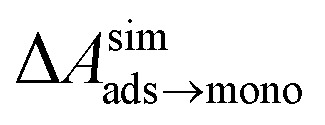
 was the average adsorbate–adsorbate energy per molecule in coarse-grained monolayers in GCMC calculations, while, for all other molecules, it was calculated from the spatial distribution of molecules in adsorbed aggregates in atomistic simulations

Molecule	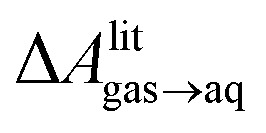 (kcal mol^−1^)	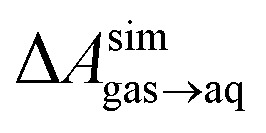 (kcal mol^−1^)	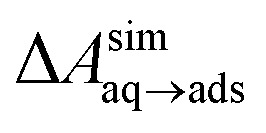 (kcal mol^−1^)	*λ*_ads_ (nm)	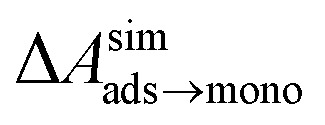 (kcal mol^−1^)	*c*_monolayer_ (μg m^−3^)
N_2_	2.43	2.94	−1.11	0.078	−2.93	1.32 × 10^12^^a^
Hexane	2.32	2.78	−4.76	0.053	−3.37	2.34 × 10^10^
Octane	2.72	3.18	−6.12	0.048	−4.22	7.91 × 10^9^
Decane	3.06	3.47	−7.84	0.045	−4.91	1.62 × 10^7^
Dodecane	3.46	3.90	−9.29	0.043	−6.01	5.03 × 10^5^
Pentadecane	4.05	4.37	−11.64	0.040	−8.12	288
Hexadecane	4.24	4.47	−12.11	0.038	−8.02	244
Octadecane	4.63	5.02	−14.34	0.035	−9.23	6.36
2-Methylheptane	2.87	3.41	−6.07	0.049	−3.93	NC^b^
Isooctane	2.89	3.02	−4.87	0.050	−4.10	NC^b^
7-Ethyltetradecane		4.81	−11.40	0.035	NC^b^	NC^b^
Ethanol	−4.99	−4.38	−2.23	0.080	−0.86	N/A^c^
Toluene	−0.77	−0.02	−5.07	0.045	−1.45	NC^b^
Limonene	0.76	0.84	−6.87	0.050	−3.26	NC^b^

a The ambient nitrogen concentration at which the dense N_2_ phase at the graphene–water interface becomes stable requires a pressure within a solid region of the N_2_ phase diagram;^38^ hence, the adsorbed N_2_ aggregate phase may not be in thermodynamic equilibrium at room temperature under any attainable conditions.

b NC: not-computed. No model was developed for grand canonical Monte Carlo simulations.

c N/A: not applicable. Ethanol does not form a 2D condensed phase (Fig. S8).

**Fig. 2:**
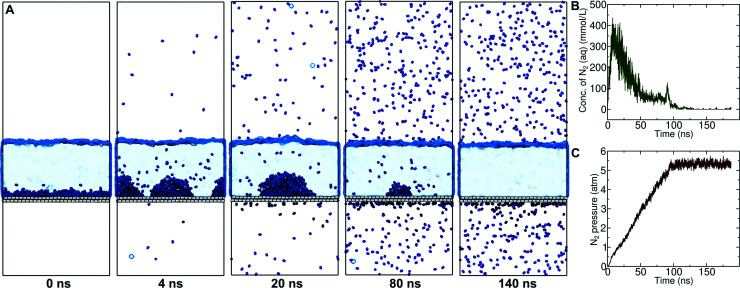
Formation and dissolution of an N_2_ aggregate at the graphene–water interface. (A) Within 4 ns, the N_2_ has coalesced into two hemispherical aggregates. Parts of one of the aggregates appear on both the left and right sides of the image, but is actually continuous owing to periodic boundary conditions. By 20 ns, the aggregates have joined into a single large bubble-like aggregate. Over the next 60 ns, N_2_ is lost from the aggregate, first dissolving into the water. These molecules can diffuse out of the aqueous phase and enter the gas phase region. Finally, all of the N_2_ molecules leave the water and enter the gas phase, consistent with the hydration free energy of N_2_ or Henry's law constant for N_2_ solubility in water. (B) Concentration of dissolved N_2_ in the aqueous phase. This value is calculated for a volume of solution far from the graphene surface, the N_2_ aggregate, and the gas–water boundary. The aqueous concentration rapidly rises as N_2_ is released from the initial interfacial *N*_2_ aggregate, but then decreases as N_2_ enters the gas phase. The minor peak in concentration at *t* = 91 ns coincides with the final disappearance of the aggregate. (C) Partial pressure of N_2_ in the gas phase region.

On the other hand, a simple approach to avoid high levels of supersaturation in simulations is to explicitly construct the system to include a gas phase volume. Then it is possible for dissolved N_2_ to diffuse out of the aqueous phase and enter the gas phase. We performed a simulation similar to that shown in Fig. S1,† but including a large gas phase region (constant volume simulation). The evolution of this system is shown in Fig. 2A. The aggregate formed as in the previous simulation; however, supersaturation was not sustained because the aqueous N_2_ could escape into the gas phase region, a process which is thermodynamically favorable. The concentration of aqueous N_2_ rose as high as 400 mmol L^−1^ during the formation of the aggregate in the first few ns of the simulation; however, this concentration dropped precipitously after about 15 ns of simulation (Fig. 2B). The reduced concentration was evidently insufficient to support the stability of the aggregate, which diminished in size, and, by 100 ns, had completely dissolved. The remaining aqueous N_2_ then entered the gas phase, after which time the aqueous phase only intermittently contained any molecules of N_2_. Fig. 2C shows the partial pressure of N_2_ in the gas phase during the same simulation. This pressure increased steadily during the simulation plateauing near 5.3 atm when most N_2_ reached the gas phase. Hence, the aggregate would be expected to be unstable for ambient N_2_ partial pressure (0.7809 atm), which is significantly lower.

We performed 9 additional simulations with increasing concentration in the N_2_ gas phase to determine what ambient N_2_ pressure would be required to stabilize an N_2_ aggregate at the graphene–water interface (Fig. S5†). The aggregate dissipated on a 100 ns timescale even with a concentration of 1.0 g mL^−1^ in the N_2_ phase, corresponding to an N_2_ pressure of 6700 atm (680 MPa). By increasing the concentration in the N_2_ phase to 1.3 g mL^−1^, we were able to observe an apparently stable aggregate; however, the associated pressure was quite extreme (2.4 GPa) and lay in solid regions of the phase diagrams for N_2_ (ref. 38) and the SPC/E water^39^ model (although both phases remained metastably fluid during the simulation). It is not clear whether the N_2_ model and SPC/E water model yield correct behavior for the aqueous solubility of N_2_ under such conditions. Nonetheless, these simulations suggest that dense N_2_ aggregates at the graphene–water interface are not thermodynamically stable at conditions anywhere near room temperature and atmospheric pressure.

### N_2_ aggregates are disordered

Neither our simulations, nor previous simulations that we are aware of,^10,25^ have suggested any long-range order in N_2_ layers at the graphene–water interface near standard conditions. This complicates the proposal that N_2_ is responsible for the striped domains observed in AFM. Fig. S3 of the ESI† shows clear long-range order in the first N_2_ layer for a liquid N_2_–water interface at 70 K. However, for a dense N_2_ layer in supersaturated water at 295 K, no such long range order is apparent for two different N_2_ models.

Based on AFM results, Teshima *et al.*^11^ proposed a structure in which gas molecules occupy the density minima between water layers at the graphene–water interface. As shown in Fig. S4 of the ESI,† our simulations predict a much different structure. Notably, the global density maxima of both N_2_ and water occur at the same distance from the surface (0.32 nm). The secondary maxima also occur at similar locations for both species (0.65 and 0.63 nm for N_2_ and water, respectively). There is no marked tendency for N_2_ to occupy the low-density region between water layers. Hence, our simulations are inconsistent with predictions of dense, ordered layers of N_2_ on graphene and intercalation between solvent layers.

### Adsorption of heavy hydrocarbons from air is thermodynamically favorable

In the typical laboratory environment, the air can act as a large reservoir of VOCs at μg m^−3^ concentrations. These molecules would be expected to dissolve into even initially pure water and contaminate the surfaces of any part of the experimental apparatus exposed to air. The molecules might then find their way to a recently cleaved graphene–water interface, either by directly diffusing through the aqueous phase, or by migrating from surfaces or air–water interfaces in contact with the graphene. Fig. 4 shows that many organic molecules exhibit enhanced densities at the air–water interface; hence, exposure of the graphene surface to this interface or movement of gas bubbles might result in deposition organic molecules on the graphene surface. Organic molecules might also leach directly into the water from polymeric materials used in the apparatus;^8^ however, contamination from these materials could, in principle, be more easily avoided than that from ambient air. Hence, here we focus on VOCs present in the air of typical indoor environments. While the precise quantities of VOCs vary considerably among different indoor environments, the major constituents are relatively consistent and often include long straight-chain alkanes.^12,13,17,40,41^

**Fig. 3:**
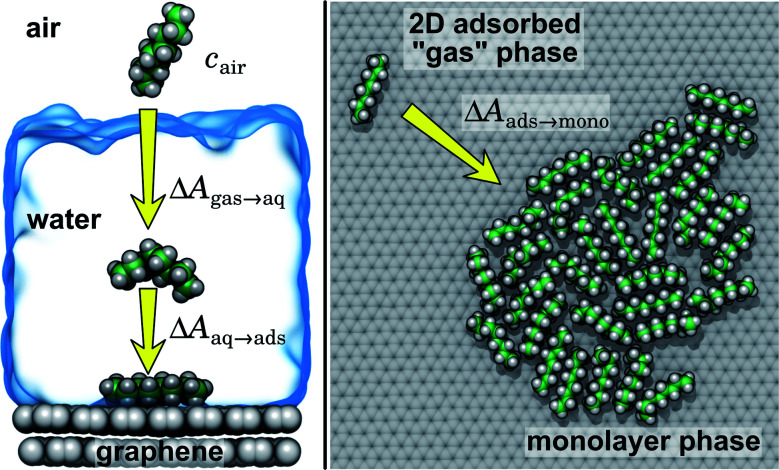
Diagram of free-energy changes for adsorption of alkanes to the graphene–water interface from air. Δ*A*_gas→aq_ is the hydration free energy. Δ*A*_aq→ads_ is the free energy for single-molecule adsorption from the aqueous phase to the graphene–water interface. Δ*A*_ads→mono_ is the free energy for condensing from the 2D gas phase to the bulk of the monolayer condensed phase.

As justified further below, we model the thermodynamics of adsorption of an organic molecule from air by four factors: (i) its concentration in ambient air (*c*_air_), (ii) its hydration free energy (Δ*A*_gas→aq_), (iii) the free energy for adsorption of a single molecule from aqueous solution to the graphene–water interface (Δ*A*_aq→ads_), and (iv) the free energy of transfer from an adsorbed (quasi-two-dimensional) gas-like phase to a condensed monolayer phase (Δ*A*_ads→mono_). Because the concentrations in air and the aqueous phase are typically low, calculations of Δ*A*_gas→aq_ and Δ*A*_aq→ads_ can be performed neglecting solute–solute and adsorbate–adsorbate interactions, while Δ*A*_ads→mono_ encapsulates the effect of adsorbate–adsorbate interactions at the interface. These four factors are diagrammed in Fig. 3.

**Fig. 4:**
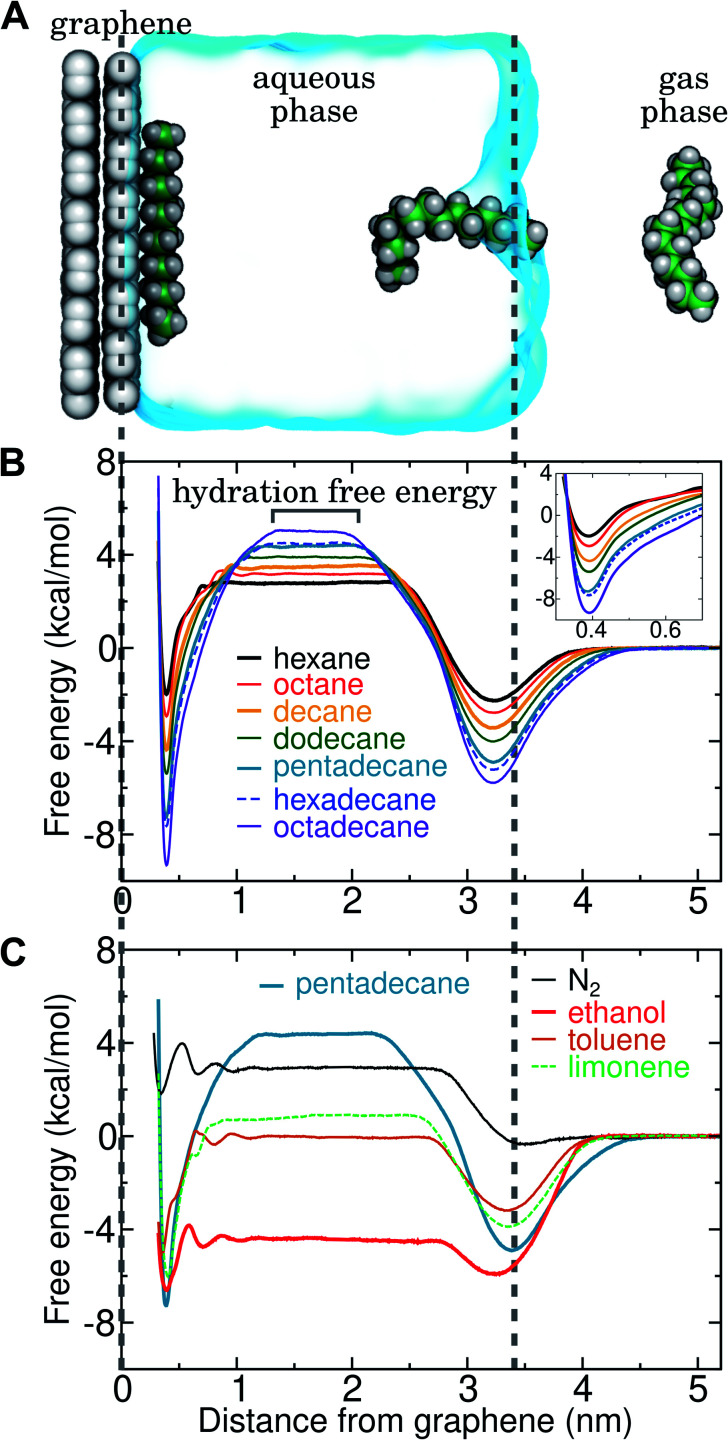
Simultaneous calculation of the free energy for hydration and adsorption at the graphene–water interface for N_2_ and several VOCs. (A) Example simulation system including graphene, water, a gas phase region, and a single molecule of pentadecane. Snapshots of the pentadecane molecule at three different times are shown. (B) Potentials of mean force as a function of distance from the graphene–water interface for a set of straight-chain alkanes. The value of this potential of mean force at a distance of 1.5 nm indicates the calculated hydration free energy for the given molecule. (Inset) Magnified view of the free energy minima at the graphene surface. (C) Potentials of mean force as a function of distance from the graphene–water interface for some common VOCs and N_2_ (using the Peng-SPCE model).

Ranges of *c*_air_ for VOCs are available in the literature.^12,13,40^ Values of Δ*A*_gas→aq_ are directly related Henry's law constants for water, which can be obtained experimentally or estimated from quantitative structure–property relationships based on experimental data.^36,37,42,43^ As shown in Fig. 4, they can also be obtained from molecular dynamics simulations. Likewise, Δ*A*_aq→ads_ can calculated from molecular dynamics simulations or obtained experimentally, although, in the latter case, it might be difficult to disentangle the effects of co-adsorbed contaminants.^19,20,44^ Finally, as described further below, we calculate Δ*A*_ads→mono_ from molecular dynamics simulations.

By including both a graphene–water and a water–air interface in the simulation system, as in Fig. 4A, we can conveniently calculate Δ*A*_gas→aq_ and Δ*A*_aq→ads_ simultaneously. We considered several VOCs having relatively large concentrations in indoor air,^12^ including C6, C8, C10, C12, C15, C16, and C18 straight-chain alkanes, as well as ethanol, toluene (an aromatic), and limonene (a terpene). Fig. 4B and C show the potentials of means for transfer from the gas phase, through the aqueous phase, to the graphene–water interface. Note that these potentials of mean force are anchored so that *w*(*z*) in the gas phase is zero. This convention is most useful when the concentration in the gas phase is known, while anchoring to the value in bulk water (in as Fig. 1) is useful when the aqueous concentration is known. The free energy at the gas–water interface, which occurs near *z* = 3.4 nm, is a local minimum for all compounds considered. This suggests that VOCs may collect at the air–water interface and transfer to graphene–water interface through contact with the former interface or bubbles present in the solution. At distances between *z* = 1.5 and 2.0 nm, the compounds are solvated in effectively bulk water. The free energy plateau in this aqueous region is therefore the hydration free energy, Δ*A*_gas→aq_ = *w*_aq_ − *w*_gas_. As is evident from Table 1, these simulation-derived Δ*A*_gas→aq_ values agree well from those derived from experiment^36,37^ or calculated using a quantitative structure–property relation.^43^ In all cases, the discrepancy is less than 0.8 kcal mol^−1^.

Except for ethanol, Δ*A*_gas→aq_ > 0, implying that the equilibrium concentration in water is lower than that in the gas phase. Owing to their hydrophobic nature, the heavy alkanes encounter large barriers to hydration, and Δ*A*_gas→aq_ becomes less favorable with carbon number (Fig. 4B). However, Δ*A*_aq→ads_ becomes favorable more rapidly with the number of carbons, meaning that the heavy alkanes show the most favorable free energies for a complete transfer from the gas phase to the aqueous phase to the graphene–water interface.

Due to its high affinity for the aqueous phase and relatively high ambient concentration compared to other VOCs, ethanol may also be present at appreciable concentrations at the graphene–water interface (Fig. 4C). Other common VOCs, such as limonene and toluene exhibit less favorable thermodynamics for transfer from air to the graphene–water interface than the heavy alkanes (Fig. 4C). N_2_ is unique among the compounds considered in that its equilibrium concentration at the graphene–water interface is lower than its associated concentration in the gas phase. However, it should be remembered that the concentration of N_2_ in air is several orders of magnitude greater than those of VOCs.

In the limit of negligible adsorbate–adsorbate interactions, the areal density at the interface can be calculated by integrating these potentials of mean force (eqn (1)). This integral,2
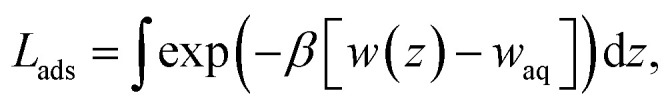
has units of length and represents the thickness of a slab of bulk solution that contains the same number of molecules as a portion of the interface with the same lateral area.^44^ When adsorption from aqueous solution is highly favorable, the limits of the integral matter little as long as they include the region around the minimum of the potential of mean force.^19^ If we define the Δ*A*_aq→ads_ as this minimum value, we can fully characterize the dilute adsorption thermodynamics by Δ*A*_aq→ads_ and a thickness *λ*_ads_ = *L*_ads_ exp(+*β*Δ*A*_aq→ads_) that represents the effective width of the free energy well (typically about half an angstrom). These values are shown in Table 1.

### Adsorption of hydrocarbons is cooperative

**Fig. 5:**
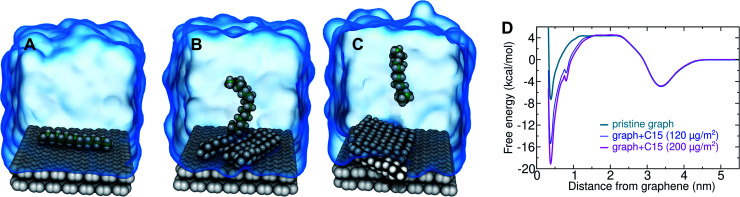
Cooperative effects on pentadecane adsorption at the graphene–water interface. (A) A single pentadecane molecule adsorbing to pristine graphene in water. (B and C) A pentadecane molecule adsorbing to a graphene–water interface with 3 or 5 other pentadecane molecules already adsorbed (having already-adsorbed pentadecane densities of 120 and 200 μg m^−2^, respectively). (D) Potentials of mean force for the systems shown in panels (A)–(C) and including graphene (*z* = 0), water (0 < *z* < 3.4 nm), and a gas phase region *z* > 3.4 nm.

Our previous computational work showed that the affinity of organic compounds for the graphene–water interface can be enhanced by the presence of co-adsorbed organic molecules.^19,20^ For instance, we found that the free energy of adsorbing an additional toluene molecule at the graphene–water interface became increasingly more favorable as the interfacial toluene density increased, until a complete monolayer was nearly formed and the favorability dropped back to near the value for the pristine surface.^20^ Cooperative adsorption can be quite complex and involve separation between two-dimensional dense and dilute phases at the interface.^45^ As shown in Fig. 5, simulations predict that the free energy for pentadecane adsorption is dramatically more favorable (ΔΔ*A*_aq→ads_ < −8 kcal mol^−1^) when the interface is already occupied by an appreciable density of pentadecane. Similar calculations (Fig.S6†) show that adsorbed N_2_ increases the affinity for adsorption of additional N_2_; however, the effect is quite weak (ΔΔ*A*_aq→ads_ = −0.9 kcal mol^−1^).

### Formation of hydrocarbon monolayers

**Fig. 6:**
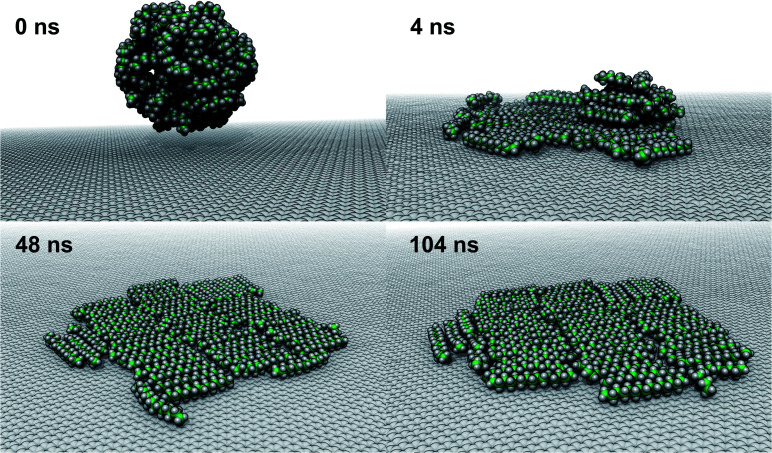
Formation of a pentadecane monolayer at the graphene–water interface. (A) Initially a droplet of pentadecane was placed above the graphene–water interface. (B–D) A partially ordered monolayer is formed within a few nanoseconds.

The cooperative adsorption of hydrocarbons is due to favorable adsorbate–adsorbate interactions, which in many cases results in the formation of a condensed monolayer phase at the graphene–water interface, as seen in Fig. 6. The free energy of transfer of isolated adsorbed molecules to the monolayer phase, Δ*A*_ads→mono_, can be calculated from the partitioning of molecules between the 2D gas phase and the 2D condensed phase in simulations of small aggregates (Fig. S8†). For the heavier alkanes, Δ*A*_ads→mono_ is so favorable that molecules never occupy the 2D gas phase on the timescale of the simulations (Δ*A*_ads→mono_ ≪ − *k*_B_*T*). In these cases, Δ*A*_ads→mono_ was calculated using the coarse-grain model described further below in this section.

Another way to characterize the tendency to aggregate is to calculate the free energy for formation of adsorbate–adsorbate pairs (Δ*A*_pair_) at the graphene surface, which we have done for all compounds as detailed in Fig. S7 and Table S1 of the ESI.† The ratio Δ*A*_aq→ads_/Δ*A*_pair_ is similar to the wetting parameter *α*_w_ defined by the Gubbins group,^46,47^ although it may not be exactly equivalent (the parameters needed to calculate *α*_w_ as defined in these papers are not directly available in our models). An important observation highlighted by calculating these ratios (Table S1†) is the effect of water: the wetting parameter Δ*A*_aq→ads_/Δ*A*_pair_ is much larger in the absence of water (graphene–gas interface) than in its presence (graphene–water interface), due to the fact that Δ*A*_aq→ads_ is more favorable in the gas phase but Δ*A*_pair_ is less favorable. As a consequence, the tendency to form condensed monolayer phases is much stronger in water than in air (as further corroborated by Fig. 11 and S14†).

**Fig. 7:**
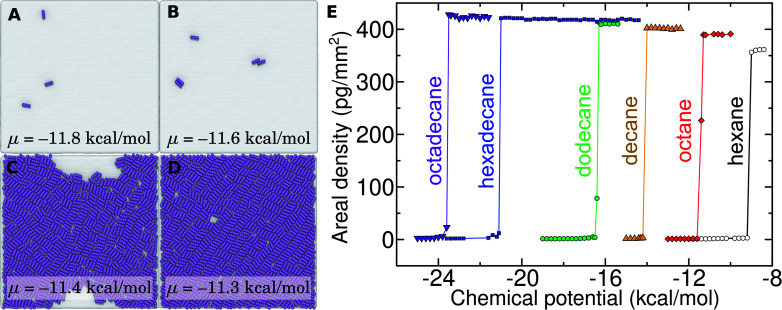
Grand canonical Monte Carlo (GCMC) calculations of alkane adsorption and monolayer formation at graphene–water interfaces. (A–D) At chemical potentials < −11.4 kcal mol^−1^, octane (represented by 4 purple coarse-grain beads) is only sparsely present at the graphene–water interface, while it forms nearly a complete monolayer at higher chemical potentials. The images show graphene patches 200 nm × 200 nm in size. (E) Mass density of alkanes at the graphene–water interface as a function of chemical potential in GCMC calculations for 6 different alkanes.

Probing the thermodynamics of adsorption at the trace concentrations of VOCs measured for indoor air (∼μg m^−3^) is not feasible with explicit atomistic simulation. Simulation systems of a typical size, *e.g.* (10 nm)^3^, would include zero VOC molecules on average in both the gas and aqueous phases for these concentrations. Therefore, we developed a novel coarse-grain model to perform constant chemical potential simulations of alkanes at the graphene–water interface using the grand canonical Monte Carlo (GCMC) method. The coarse-grain model (implicit-solvent) was explicitly constructed to reproduce the adsorption free energy from air (Δ*A*_gas→aq_ + Δ*A*_aq→ads_) calculated in our explicit-solvent atomistic simulations (Fig. 4). The alkanes were represented as a rigid chain of beads, with one bead for every two carbon atoms. Their interaction was calibrated to reproduce the free energy of adding a small alkane (hexane or octane) to its monolayer phase (see Fig. S9† and Methods). In these GCMC calculations, we observed dramatic changes in the coverage of the interface as a function of chemical potential, as shown in Fig. 7. For example, at chemical potentials of *μ* = −11.8 and −11.6 kcal mol^−1^, only isolated octane molecules or small octane clusters are observed (Fig. 7A and B). However, at a slightly greater chemical potential, the interface rapidly fills with a nearly complete octane monolayer (Fig. 7C and D). We therefore are able to estimate a critical chemical potential above which a dense monolayer forms and below which the adsorbed alkanes behave as a 2D gas. This chemical potential can be directly related to the gas phase concentration and, therefore, experimental data on VOC concentrations in indoor air. We have included this estimated critical gas phase concentration (*c*_monolayer_) in Table 1.

Of relevance for graphitic carbon materials in the presence of typical indoor air, we predict that an octadecane concentration of 6 μg m^−3^ in air may be sufficient for a complete octadecane monolayer to occupy the graphene–water interface at thermodynamic equilibrium. Hexadecane is predicted to completely cover the interface at a somewhat higher, but still trace, concentration (244 μg m^−3^). It should be noted that larger concentrations of these alkanes have been measured in some indoor environments, including octadecane concentrations as high as 41 μg m^−3^ and hexadecane concentrations of nearly 300 μg m^−3^.^40^ Such concentrations of heavy organic compounds, which could be present in labs for various reasons, likely result in complete contamination of initially clean graphene–water interfaces. These results may explain the difference in AFM force profiles in water between freshly cleaved graphite and graphite exposed for 30 minutes to ambient air.^26^

### Trends in the adsorption thermodynamics

**Fig. 8:**
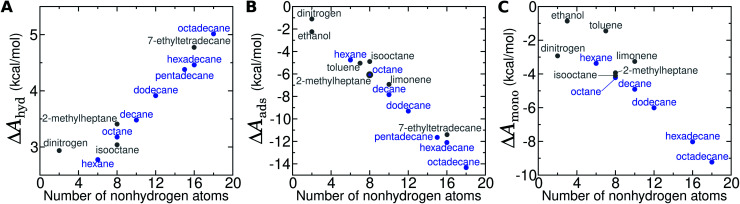
Adsorption thermodynamics at the graphene–water interface for VOCs and N_2_. (A) Hydration free energy (Δ*A*_gas→aq_) as a function of the number of heavy atoms. Straight-chain alkanes are indicated in blue, while other compounds, including branched alkanes are indicated in gray. For legibility, ethanol, toluene, and limonene have been left outside of the range this graph due to their much more favorable Δ*A*_gas→aq_ values. (B) Free energy of adsorption to the graphene–water interface from aqueous solution (Δ*A*_aq→ads_) as a function of the number of heavy atoms. (C) Free energy of transfer from the adsorbed 2D gas phase to the adsorbed monolayer phase (Δ*A*_ads→mono_) as a function of the number of heavy atoms.

For the straight-chain alkanes, there are clear trends in Δ*A*_gas→aq_, Δ*A*_aq→ads_, and Δ*A*_ads→mono_ with molecular mass (Fig. 8). In particular, the hydration free energy Δ*A*_gas→aq_ increases approximately linearly with the number of carbon atoms and remains within a much smaller range (2.8–5.0 kcal mol^−1^) than Δ*A*_aq→ads_ and Δ*A*_ads→mono_. Therefore, despite increasingly unfavorable hydration, adsorption and monolayer formation becomes more favorable for longer straight-chain alkanes. Indeed, both Δ*A*_aq→ads_ and Δ*A*_ads→mono_ appear to decrease (become more favorable) superlinearly with alkane molecular mass. Intermolecular interaction of N_2_ is very weak (as is clear from its boiling point of 77 K), which explains much about its transfer free energies. For N_2_, Δ*A*_gas→aq_ is unfavorable and lies between that of hexane and octane, which is mostly due to its disruption of water structure (loss of configurational entropy for the water coordinating the solute with no energetic compensation^48^). The orientational order of water is also increased at its interfaces with graphene, as well as with alkane monolayers (Fig. S11†). Even in the dense N_2_ aggregate at the graphene–water interface, intermolecular interactions appear to be weak: Δ*A*_ads→mono_ is very similar in magnitude to Δ*A*_gas→aq_.

Compared to straight-chain alkanes with similar molecular masses, ethanol, toluene, and limonene exhibit much more favorable hydration (Table 1) but less favorable monolayer formation (Fig. 8C). The differences between branched and straight-chain alkanes appear more complex and are discussed further below (Fig. 11 and S12†).

### Alkane mixtures

**Fig. 9:**
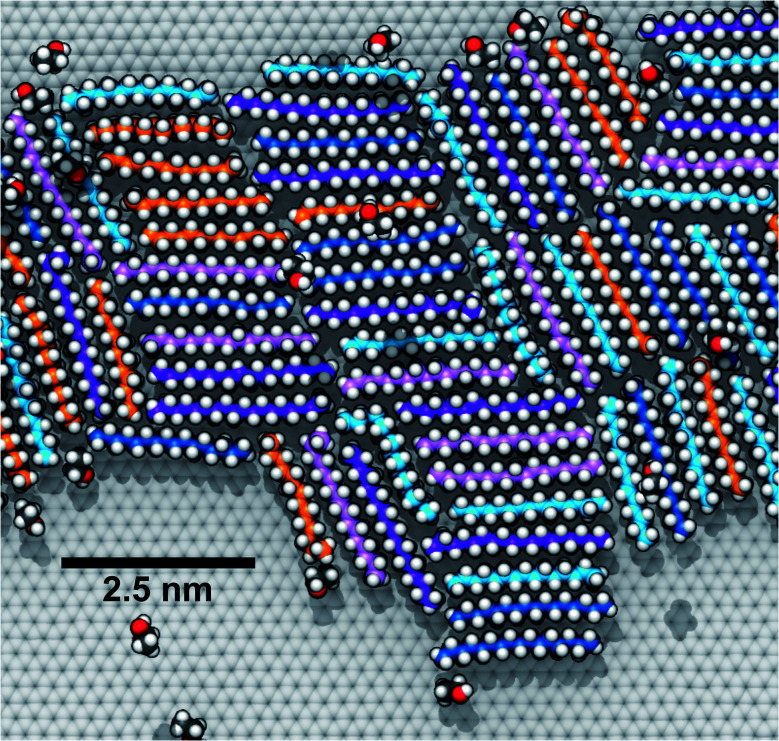
Arrangement of a mixture of heavy hydrocarbons (C14–C18) and ethanol at the graphene–water interface. Tetradecane (orange C), pentadecane (teal C), hexadecane (blue C), heptadecane (pink C), octadecane (purple C), and ethanol (dark gray C).

To explore how a mixture of hydrocarbons might behave at the graphene–water interface, we performed a simulated annealing calculation including equal numbers of each of the C14–C18 straight-chain alkanes and ethanol. The molecules were initially randomly distributed within the water phase, but all of the alkanes rapidly adsorbed to the graphene–water interface and remained bound. Fig. 9 shows that the alkanes lie flat on the interface, maximizing their contact with graphene, and adopted mostly straight conformations. Significant long-range order is apparent, characterized by rows of alkane molecules with similar orientations, which remained fairly stable throughout the room-temperature portion of the simulation. The ethanol molecules, on the other hand, did not form a permanent part of the structure and rarely stayed in one location for long. The alkanes tend to align along the zigzag axes of the graphene, which is consistent with structures observed in AFM.^8^ The gaps between the rows of molecules and the boundaries between domains of different orientations could be responsible for the observed stripe-like patterns, with the pitch of stripes depending on the length of the molecules. Observed patterns have shown pitches from 2 to 8 nm,^8,9,23^ which, if due to rows of straight-chain alkanes, could correspond to molecules ranging from pentadecane (2 nm length) to alkanes of more than 60 carbons. It is possible that observed stripe-like patterns consist of different molecules depending on the conditions of the experiment: while they may consist of airborne hydrocarbons in some experiments, distinct molecules, such as PDMS oligomers,^22^ might predominate in others.

### Potential explanation for observed “nanopancakes”

**Fig. 10:**
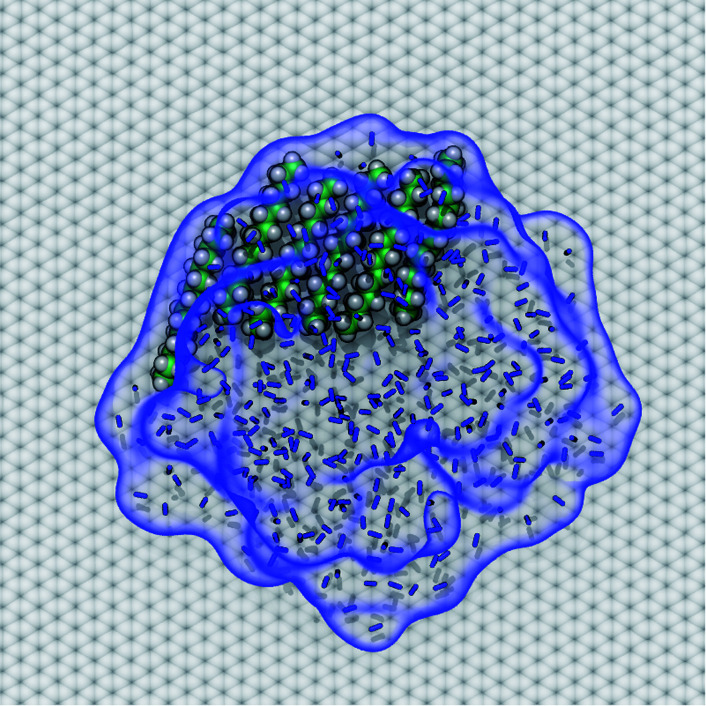
A cluster of pentadecane that spontaneously coalesced with an N_2_ aggregate and occupies part of the interface between the N_2_ aggregate and graphene surface. The N_2_ molecules are represented by blue bonds. The interface between the N_2_ aggregate and water is highlighted by a transparent blue surface, to more clearly show its outline.

AFM studies have observed curious structures referred to as “nanopancakes” or “micropancakes”—dense spots that appear inside bubble-like objects that form under conditions of N_2_ supersaturation.^49,50^ Our simulations (S1 and S10) and those of others^10,25^ support the existence of such N_2_ aggregates under supersaturated conditions. Furthermore, as shown in Fig. S10,† we find that heavy hydrocarbons, such as pentadecane, are attracted to N_2_–water interfaces and insert between the N_2_ aggregates and graphene. Hence, we propose that the dense spots might be clusters of hydrocarbons embedded at the bottom of N_2_ aggregates or bubbles. To explore this hypothesis, we performed a simulation of a graphene–water–N_2_ system including a few randomly placed pentadecane molecules. After several nanoseconds, the pentadecane molecules merged with the N_2_ aggregates and formed clusters at the N_2_–water interface, in an arrangement reminiscent of “nanopancake” images (Fig. 10).

### Branched alkanes

**Fig. 11:**
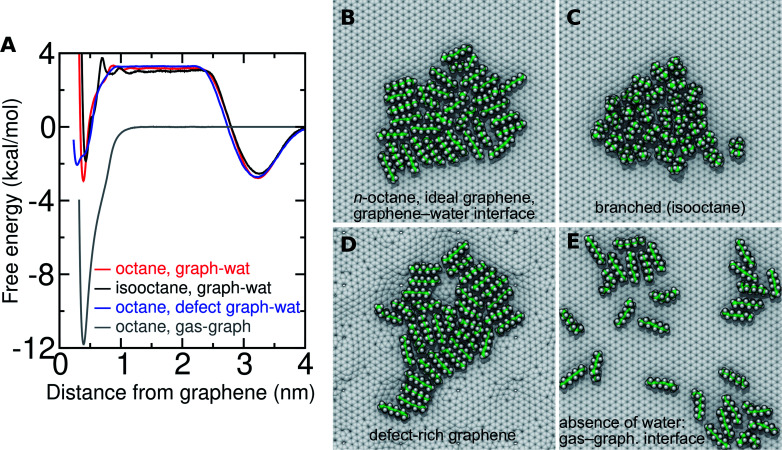
Adsorption thermodynamics of 8-carbon alkanes on graphene under varied conditions. (A) Free energy for transfer of 8-carbon alkanes from the gas phase to the graphene surface, considering the difference between a straight-chain alkane and a branched alkane (2,2,4-trimethylpentane also known as isooctane), an ideal graphene structure and defect-rich graphene, and a graphene–gas interface (in the absence of water). (B) Aggregate of *n*-octane at an ideal graphene–water interface. (C) Aggregate of isooctane at an ideal graphene–water interface. (D) Aggregate of *n*-octane at the aqueous interface of defect-rich graphene. (E) Aggregate of *n*-octane at a graphene–gas interface.

In the real world, VOCs typically consist of a wide of variety of chemical species, including branched alkanes as well as straight-chain isomers.^40^Fig. 11A shows the free energy for transfer from the gas phase to the graphene–water interface for *n*-octane and the octane isomer 2,2,4-trimethylpentane (isooctane). In the simulations, adding branches to the alkanes appears to have non-monotonic effects on Δ*A*_gas→aq_ (Table 1); the experimental rankings vary among sources, so whether this is true in reality remains unclear.^37^ There is a more clear effect on Δ*A*_aq→ads_: for the straight-chain alkanes all hydrocarbon groups usually make direct contact with the graphene surface; however, in isooctane, it appears impossible for all groups to contact the surface at once due its tertiary carbon. This makes adsorption substantially less favorable (Fig. 11A). As shown in Table 1 and Fig. S12,† the adsorption affinity is also reduced for 7-ethyltetradecane in comparison to its straight-chain analog, hexadecane, resulting in a Δ*A*_aq→ads_ similar to the straight-chain alkane with one fewer carbon atom. Furthermore, aggregates of straight-chain alkanes at the graphene–water interface, such as *n*-octane (Fig. 11B), show a clear tendency for alignment of neighboring molecules. Branched isomers isooctane (Fig. 11C) and 2-methylheptane (Fig. S12†) exhibit less order and formation of pairs is less favorable (ΔΔ*A*_pair_ = 1.7 kcal mol^−1^). However, the difference in Δ*A*_ads→mono_ between *n*-octane and isooctane is not as dramatic as would be expected from this difference in Δ*A*_pair_ (Fig. S12†) because the more compact structure of isooctane means that it has more neighbors in the monolayer phase (typically 5–7), as is evident in Fig. 11C.

### Graphene defects

Real graphene is not perfectly crystalline, but includes defects, such as the common Stone–Wales defects. To study the effect of defects on adsorption to the graphene–water interface, we made use of a defect-rich graphene structure generated in previous work^20^ by ReaxFF^51^ simulations, which contains a ratio of 61 : 10 : 8 : 1 of 6-, 5-, 7-, and 8-membered carbon rings. As in our previous work,^20^ we find that the defects introduce undulations in the graphene sheet and that organic molecules exhibit a preference for concave regions over convex regions. This is reflected in the free energy profile, which has a broader minimum at a distance of *z* = 0.29 nm from the graphene center of mass rather than at *z* = 0.39 nm for flat graphene (Fig. 11A and S13†). Overall, the adsorption affinity is reduced compared to flat graphene. For octane, *L*_ads_ = 4.4 nm for defect-rich graphene and *L*_ads_ = 7.2 nm for ideal graphene. The effect is even more pronounced for pentadecane (Fig. S13†), *L*_ads_ = 1900 and 9600 nm, respectively. Fig. 11D shows octane filling a valley on the defect-rich graphene, while a small protuberance remains bare. Defects appear to disrupt the order of the monolayer and likely somewhat reduce the favorability of monolayer formation. On the other hand, the concave regions have a higher adsorption affinity than flat graphene^20^ and may serve as locations from which monolayers can nucleate.ftab

### Behavior in the absence of water

While this paper focuses on the graphene–water interface, it is instructive to compare these results with those at the graphene–gas interface. The adsorption of alkanes at the graphene–gas interface is much more favorable than at the graphene–water interface (Fig. 11A); however, the tendency to aggregate and the cohesion of the aggregate is much reduced (Fig. 11E). For example, octane shows an adsorption free energy of −11.8 kcal mol^−1^ at the graphene–gas interface, which is much more favorable than its Δ*A*_aq→ads_ (−6.1 kcal mol^−1^) or the full free energy for transfer from the gas phase to the graphene–water interface (Δ*A*_gas→aq_ + Δ*A*_aq→ads_ = −2.9 kcal mol^−1^). While octane molecules rapidly coalesce into a single monolayer aggregate at the graphene–water interface (Fig. 11B), association between octane molecules is much looser at the graphene–gas interface. This is reflected by the reduced favorability of Δ*A*_pair_ at the graphene–gas interface (−1.0 and −3.8 kcal mol^−1^ in the absence and presence of water, respectively). As detailed in Fig. S14,† these trends are also followed for pentadecane and N_2_. Instead of forming a roughly disc-shaped monolayer phase, as it does at the graphene–water interface, pentadecane appears to form filaments of aligned molecules in the absence of water (Fig. S14H†).

### Hydrocarbon model agrees well with AFM force profiles

**Fig. 12:**
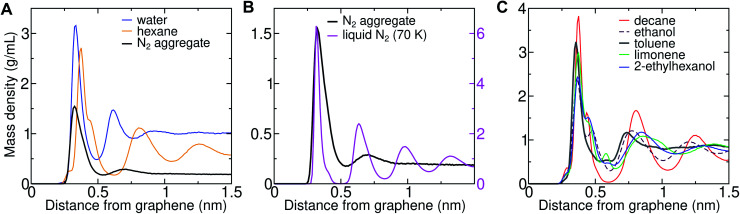
Density profiles for solvent–graphene systems. (A) Mass density as a function of distance from graphene–water and graphene–hexane interfaces, as well as a graphene–water interface including an N_2_ aggregate (using the Peng-SPCE model). (B) Similar plot including the mass densities of the N_2_ aggregate and liquid N_2_ at 70 K using the same N_2_ model. To facilitate comparison, the aggregate and liquid N_2_ are plotted with different scales (the left and right scales, respectively). (C) Mass density profiles for other common VOCs.

Three-dimensional AFM shows oscillations in the force as a function of distance from solid–solvent interfaces.^6,26^ It was observed that the force profile showed a larger distance between consecutive maxima on graphene (0.43–0.53 nm) than on mica (0.34 nm). Molecular dynamics simulations can help in interpreting the link between these oscillations and the atomic structure at the interface. We previously demonstrated that the wavelength of force undulations experienced in simulations by a model AFM tip asperity agree well with the mass density undulations of the solvation layers.^26^ We find that solvating graphene with compounds of different chemical natures leads to distinct density oscillations, reflecting the existence of discrete solvation layers above the graphene surface (Fig. 12). As is evident in Fig. 12A, solvation layers of water have the smallest separation between density peaks, followed by N_2_, while alkanes exhibit significantly larger wavelengths (Fig. 12A and B). As shown in Fig. 12B, the locations of the mass density peaks for the N_2_ aggregate are only slightly farther out than those for liquid N_2_ at 70 K, although the latter are much more pronounced.

The peak-to-peak distances in the mass density profiles of water, N_2_, hexane, and several common VOCs are shown in Table 2. Hexane shows a characteristic wavelength of 0.43–0.45 nm, in agreement with the distance between force maxima in AFM for a graphite–hexane system (0.44–0.52 nm).^26^ Larger straight-chain alkanes exhibit the same wavelength in density undulations on graphene, but the amplitude of these undulations increases with molecular mass. Aggregates of N_2_ in water and cryogenic liquid N_2_ show wavelengths that are appreciably shorter (0.32–0.38 nm) than the alkanes and are somewhat longer than a pure graphene–water interface (0.28–0.33 nm), but significantly shorter than those measured for systems nominally consisting of graphene–water and graphite–water.^6,26^ Taken together, the simulation and AFM data are consistent with alkane-like molecules occupying the graphene–water interface, but inconsistent with N_2_ layers or simply water.

**Table 2:** Distances between consecutive peaks in the mass density profiles of solvents on graphene

Solvent	Δ*z*_1_ (nm)	Δ*z*_2_ (nm)	Δ*z*_3_ (nm)
Water	0.28	0.31	0.33
N_2_ aggregate	0.32	0.38	0.34
N_2_ (liquid, 70 K)	0.31	0.32	0.35
Hexane	0.43	0.45	0.45
Decane	0.43	0.45	0.44
Ethanol	0.41	0.43	0.40
Toluene	0.38	0.20	0.45
2-Ethylhexanol	0.48	0.47	0.41
Limonene	0.48	0.53	0.47

In some cases, it might be possible to exclude certain VOCs from being major components of the interfacial contaminants based on the AFM data (Fig. 12C). Aromatics like toluene exhibit smaller distances between their first two density peaks than the experimental force undulations and, therefore, are unlikely to be major components of the interfacial contaminant layer. Ethanol, which is one of the highest-concentration VOCs in typical indoor air, also exhibits a somewhat shorter wavelength than the measured undulations. Branching of the alkanes increases the peak-to-peak distance (2-ethylhexanol), but the values are still within the experimentally measured range. The cyclic terpene limonene also exhibits a larger wavelength than straight-chain alkanes, but this wavelength remains near the experimental range.

## Conclusion

The conclusion of this work is that the striped patterns observed by AFM at the graphene–water interface are likely due to ordered arrangements of hydrocarbons, such as alkanes, that migrate to the interface from the air or from the surfaces of materials used in the experiments. Sufficient concentrations of these molecules for accumulation at the graphene–water interface can be present in indoor air. Our grand canonical Monte Carlo calculations predict that, beyond a critical ambient concentration, the alkane aggregates nucleate at the graphene–water interface and grow into complete monolayers, driven by highly favorable adsorbate–adsorbate interactions. For heavy straight-chain alkanes such as octadecane, concentrations on the order of a few μg m^−3^, which are less than some measured values for indoor air, are sufficient for the complete monolayer to become the thermodynamic equilibrium. The characteristic spacing between layers of straight-chain or branched alkanes on graphene perpendicular to the surface agrees well with the characteristic distance between extrema in force profiles measured by AFM for systems that are nominally graphene immersed in water. Hence, we propose that a graphene surface covered by a monolayer or multilayer of heavy alkanes might provide a representative model for experiments on graphene immersed in water.

We find no evidence of ordered layers of N_2_ on graphene in water at room temperature. They seem unlikely owing to the weak affinity of N_2_ for the graphene–water interface and fairly weak cooperative interactions between N_2_ (as evidenced by its low boiling point). The simulations predict that aggregates of N_2_ can form in highly supersaturated aqueous solutions of N_2_, but dissolve if the solution ceases to be supersaturated. The (meta)stability of these aggregates in simulation depended on the model of N_2_ used; hence, more work should be done to validate and perhaps improve existing models of N_2_ for this type of study.

The results here, highlighting the importance of adsorbed organic compounds at solid–water interfaces, are likely to apply not only to graphene, but also to other graphitic materials such as graphite and carbon nanotubes, as well as other hydrophobic surfaces, such as 2D metal dichalcogenides^6^ and synthetic polymers. In the latter case, adsorbed hydrocarbon layers would likely be more difficult to image owing to rougher topography. Very hydrophilic materials, such as mica, appear not to accumulate such layers under typical laboratory conditions, as their force profiles^6^ are consistent with a simple model including only mica and water. Simulations also show a low affinity of hydrocarbons for mica.^26^ This work should further our understanding of the physical and chemical properties of interfaces exposed directly or indirectly to atmospheric air.

## Methods

### Molecular dynamics protocols

Simulations were performed using protocols similar to previous work.^19,20^ All simulations were performed with NAMD 2.14 (ref. 52) using a 4 fs timestep^53^ and particle-mesh Ewald electrostatics.^54^ Lennard-Jones forces were smoothly truncated from 10 to 12 Å. Except where otherwise noted, the temperature was maintained at 295 K by a Langevin thermostat^55^ using a 1 ps^−1^ damping constant and the pressure was maintained at 1.01325 bar using the Langevin piston algorithm with the system dimensions adjusted independently along all three dimensions.^56^ Periodic boundary conditions were applied along all three axes, making the graphene patch form an effectively infinite surface in the *xy* plane. To represent a mounted sample, the atoms of the lower sheet were restrained to their initial *z* positions by 1 kcal mol^−1^ Å^−2^. Solutes interacted only with the unrestrained upper layer of graphene. Each system underwent 2000 steps of energy minimization before beginning production runs.

### Molecular dynamics force fields

Except for simulations denoted Peng-SPCE, water was represented by the modified TIP3P model of the CHARMM force field and graphitic carbon was represented by the CG2R61 type of the CHARMM General Force Field.^35^ This combination has yielded good agreement with experiment in previous work.^19,20^ For the Peng-SPCE model, we sought to match Peng *et al.*^25^ as much as possible by using the SPC/E water model^57^ and the graphitic carbon Lennard-Jones parameters *ε* = 0.0936902 kcal mol^−1^ and *R*^min^ = 3.58065 Å. In all cases, the bonded parameters for graphitic carbon came from the CHARMM General Force Field.^35^ All organic compounds were represented by the CHARMM General Force Field, version 4.3.^35^ Parameters for limonene were generated using the CGenFF web interface.^58,59^ For dinitrogen, we used a variety of nonbonded parameters, given in Table 3, with Lennard-Jones energies given by *V*^LJ^_*ij*_ = *ε*_*ij*_([*R*^min^_*ij*_/*r*_*ij*_]^12^ − 2[*R*^min^_*ij*_/*r*_*ij*_]^6^), where *r*_*ij*_ = |**r**_*j*_ − **r**_*i*_| is the distance between the two atoms. Lorentz–Berthelot combining rules are used for all Lennard-Jones interactions. The bond parameters for N_2_ were taken from Sharma and Adhikari,^60^ with *V*^bond^_*ij*_ = *K*_b_(|**r**_*j*_ − **r**_*i*_| − *b*)^2^, where *K*_b_ = 1649.1396 kcal mol^−1^ Å^−2^, *b* = 1.0975 Å.

**Table 3:** Non-bonded parameters used for simulations of dinitrogen–graphene–water systems

N_2_ model	*ε*_N_ (kcal mol^−1^)	*R* ^min^_N_ (Å)	*q*_N_ (*e*)	*q*_m_ (*e*)	*ε*_C_ (kcal mol^−1^)	*R* ^min^_C_ (Å)	H_2_O model
Jiang^33^	0.0723338	3.72657	−0.482	0.964	0.07000	3.9848	TIP3P
Vujić^34^	0.0799646	3.72657	−0.482	0.964	0.07000	3.9848	TIP3P
Bouanich^32^	0.0739235	3.69464	0.000	—	0.07000	3.9848	TIP3P
Peng-TIP3P^25^	0.0690000	3.66035	0.000	—	0.07000	3.9848	TIP3P
Peng-SPCE^25^	0.0690000	3.66035	0.000	—	0.09369	3.5807	SPC/E

### Free energy calculations

Potentials of mean force (PMFs) were calculated using the adaptive biasing force (ABF) method.^61,62^ For calculating *w*(*z*), the transition coordinate was defined as the vector from the center of mass of upper layer of graphene to the center of mass of the solute, projected onto the axis perpendicular to the graphene sheets (the *z* axis). To calculate Δ*A*_pair_, the transition coordinate was the distance between the two adsorbate molecules projected into the plane parallel to the graphene, 
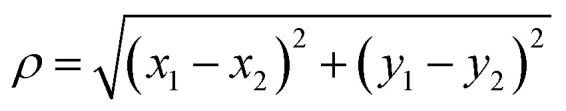
. The transition coordinate grid size was 0.05 Å. To obtain high precision, all ABF simulations were run for 1–2 μs of simulated time.

### Model for N_2_ adsorption

The system shown in Fig. 1 contains two rectangular patches of graphene totaling 672 carbon atoms (average dimensions of 29.4 Å × 29.7 Å). A molecule of N_2_ was placed on the top layer of graphene and the entire system was solvated with 797 water molecules.

### Model for gas–water–graphene calculations

The systems for simulating the adsorption and hydration of organic compounds at the graphene–water and water–gas interfaces (Fig. 4 and 5) were built using the same two layers of graphene (29.4 Å × 29.7 Å), one solute molecule (hexane, decane, pentadecane, hexadecane, octadecane, N_2_, ethanol, toluene, or limonene) and 847 molecules of water. The size of the system along the *z*-axis was 120 Å. The simulations were performed at constant volume to maintain the gas phase region. A potential energy barrier was placed at *z* = 30 Å using the grid force feature of NAMD^63^ to prevent vapor phase water molecules from adsorbing to the lower graphene layer.

### Other models

Self-assembly of the alkane–ethanol mixture (Fig. 9) was studied using a system containing two larger layers of graphene (117.4 Å × 118.6 Å), 486 ethanol molecules, 54 molecules for each C14–C18 straight-chain alkane, and 30 874 water molecules. The system was held at 500 K for with fixed system volume for 20 ns, followed by cooling to 295 K over 10 ns. The simulation was then continued at 295 K for 155 ns under constant pressure conditions (with a barostat applied).

The formation of N_2_ aggregates (Fig. S1†) was simulated by randomly placing 512 N_2_ molecules at the graphene–water interface using two graphene layers with dimensions of 115.8 Å × 116.9 Å and 24 758 water molecules. The Peng-SPCE model was used and simulation was run at constant pressure for 200 ns. The simulation detailed in Fig. 2 was performed with the same initial positions of the atoms, but at constant volume with a large gas phase region (the *z*-dimension totaled 300 Å).

Calculations of Δ*A*_pair_ were performed in systems 50.8 × 48.9 × 23.5 Å^3^ and run for *t* > 1 μs. The simulation detailed in Fig. 6 used two 150.3 Å × 144.5 Å graphene layers and included 53 molecules of pentadecane and 62 120 molecules of water. Simulations of VOC aggregates (such as those shown in Fig. 11 and S8†) were performed with 32 octane molecules or a similar mass of other VOCs. The systems measured 101.7 × 97.8 × 29.6 Å^3^. These systems were run for *t* > 1 μs.

Fig. 12 was produced using systems containing two layers of graphene (29.4 Å × 29.7 Å). For each solvent, the systems were solvated with PackMol^64^ with a number of molecules (water, 1547; hexane, 214; decane, 143; ethanol, 478; limonene, 172; 2-ethylhexanol, 79; liquid N_2_, 865) sufficient to obtain a system *z*-dimension of about 60 Å. The N_2_ aggregate system included two layers of graphene of dimensions 115.8 Å × 116.9 Å, 3072 N_2_ molecules, and 35 008 water molecules. This simulation was performed at constant volume (*z*-dimension 130 Å) to prevent the N_2_ from forming an extended gas phase.

### Grand canonical Monte Carlo

The grand canonical Monte Carlo (GCMC) method,^65^ as implemented in the program LAMMPS^66^ (version 29Oct20), was employed to simulate alkane–water–graphite systems at constant alkane chemical potential. To facilitate insertion and deletion of molecules (as required by the GCMC method) and allow for large systems, we developed an implicit-solvent coarse-grain representation of alkanes and graphene–water interface, which is described in the next paragraph. The systems had square geometries in the *xy*-plane ranging from (200 Å)^2^ to (800 Å)^2^ and were periodic these directions, while having fixed boundaries in the *z*-direction. The GCMC calculations were a hybrid of GCMC and molecular dynamics simulation, with 25 GCMC insertion or deletion steps attempted every 50 dynamics steps. The molecular dynamics used a timestep of 1 fs to accommodate poorly equilibrated inserted molecules. The alkane molecules were fully rigid during all steps. Each system was run for 200 ns of simulated time. Because the GCMC insertion algorithm of LAMMPS applies a random rotation to the molecules about their center of mass and the molecules consisted of multiple beads, it was necessary to modify the LAMMPS code so that the algorithm did not place coarse-grained beads beyond the wall. The modified C++ source file, fix_gcmc.cpp, is included in the Zenodo repository (see “Data and software availability”).

### Coarse-grain models

In the GCMC calculations, each alkane molecule was represented as a rigid rod consisting of spherical particles (beads), with one bead per two carbon atoms. The mass of all beads was 28.6 Da, approximately representing two CH_2_ or CH_3_ groups. Like hexadecane, pentadecane was represented with 8 beads, but the representation of the surface differed between the two molecules (Table 4). Consistent with the average structure in atomistic simulations of alkanes at the graphene–water interface, the coarse-grain models of the straight-chain alkanes were assigned straight structures with 2.55 Å between beads. The graphene–water interface was emulated by a 12–6 Lennard-Jones potential energy function *E*_wall_(*z*) = 4*ε*_wall_[(*σ*_wall_/*z*)^12^ − (*σ*_wall_/*z*)^6^], that was applied to the coarse-grain beads, using the “wall/lj126” feature of LAMMPS. This function yielded a better fit to *w*_air–water–graph_ than the other alternative, a 9–3 potential. The parameters of *E*_wall_(*z*) were chosen to mimic the potentials of mean force calculated from the atomistic models, the gas–water–graphene PMFs (*w*_air–water–graph_(*z*), shown in Fig. 4), so that the chemical potentials of the GCMC method could be equated with concentrations in ambient air. The depth of the energy well was set to the minimum of the PMF at the graphene surface divided by the number of beads per molecule, *ε*_wall_ = *w*^min^_air–water–graph_/*B*, because the PMF was calculated for the center of mass of the entire molecule. The width of the energy well was chosen so that the shape near the minimum was similar between *w*_air–water–graph_(*z*) and the *E*_wall_. Specifically, we optimized *σ*_wall_ to produce the same value of the thermodynamic adsorption parameter: 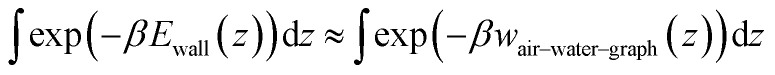. The exponential in the integrand ensures that the contributions to integral come principally from the region within a short distance of the PMF minimum. The integral was truncated at 6 Å to exclude contributions from the air–water interface, which would be significant for hexane and octane. An additional harmonic wall was placed beyond the minimum of *E*_wall_(*z*) (at *z* = 2^1/6^*σ*_wall_ + 1.8 Å) to capture the greater sharpness in the minimum of the PMF relative to the 12–6 potential. This potential yielded a good match with the *z*-distribution of atoms (converted to beads) from atomistic simulations of alkane aggregates at the graphite–water interface (Fig. 4).

**Table 4:** Parameters for coarse-grain models of alkanes at the graphene–water interface used in the simulations

Molecule	Beads (per mol.)	*ε*_wall_ (kcal mol^−1^)	*σ*_wall_ (Å)	*ε*_bead_ (kcal mol^−1^)	*σ*_bead_ (Å)
Hexane	3	0.661	2.102	0.86	3.795
Octane	4	0.734	2.684	0.86	3.795
Decane	5	0.873	3.321	0.86	3.795
Dodecane	6	0.911	3.634	0.86	3.795
Pentadecane	8	0.908	3.968	0.86	3.795
Hexadecane	8	0.955	3.873	0.86	3.795
Octadecane	9	1.036	4.054	0.86	3.795

Inter-bead interactions, which captured the intermolecular interactions of the alkanes, were also of the 12–6 Lennard-Jones type and parameterized by comparing atomistic simulations of hexane, octane, and decane aggregates at the graphene–water interface to multiple coarse-grain models. Fifty different parameter sets (*ε*_bead_ and *σ*_bead_) were tried for *ε*_bead_ in the range [0.4, 1.0] kcal mol^−1^ and *σ*_bead_ in the range [3.74, 4.187] Å. As shown in Fig. S9 of the ESI,†*ε*_bead_ = 0.86 kcal mol^−1^ and *σ*_bead_ = 3.795 Å yielded good agreement with the atomistic simulations for the cylindrical radial PMFs of the aggregates. The details of the coarse-grain model are summarized in Table 4.

## Data and software availability

The simulation data described in this work are freely available for download from Zenodo (https://doi.org/10.5281/zenodo.6050816). The archive includes files needed to run the simulations described here using NAMD and LAMMPS, as well as the output of the simulations and analysis scripts. The files are organized into directories corresponding to the figures of the main text and ESI.† They include molecular model structure files (in CHARMM/NAMD psf format), force field parameter files (in CHARMM format), initial atomic coordinates (pdb format), NAMD or LAMMPS configuration files, Colvars configuration files, NAMD log files, and NAMD output including restart files (in binary NAMD format) and trajectories in dcd format (downsampled due to space constraints). Analysis is controlled by shell scripts (Bash-compatible) that call VMD Tcl scripts. A modified LAMMPS C++ source file is also included. The programs VMD, NAMD, and LAMMPS are distributed under open source licenses and are free for academic use.

## Author contributions

Ravindra Thakkar: investigation, writing – review & editing. Sandun Gajaweera: investigation. Jeffrey Comer: conceptualization, methodology, software, investigation, funding acquisition, writing – original draft, writing – review & editing, supervision, visualization.

## Conflicts of interest

There are no conflicts to declare.

## Supplementary Material

NA-004-D1NA00570G-s001
