# Supplementary material for: Organic contaminants and atmospheric nitrogen at the graphene–water interface: a simulation study
Source: Nanoscale Adv. 2022 Mar 16;4(7):1741–57. doi: 10.1039/d1na00570g (PMC9417612; doi:10.1039/d1na00570g)
Supplement: NA-004-D1NA00570G-s001 [file NA-004-D1NA00570G-s001.pdf]

Electronic Supplementary Material (ESI) for Nanoscale Advances.
This journal is © The Royal Society of Chemistry 2022

Supplementary Information for

Organic contaminants and atmospheric nitrogen at the graphene–water interface: A simulation study

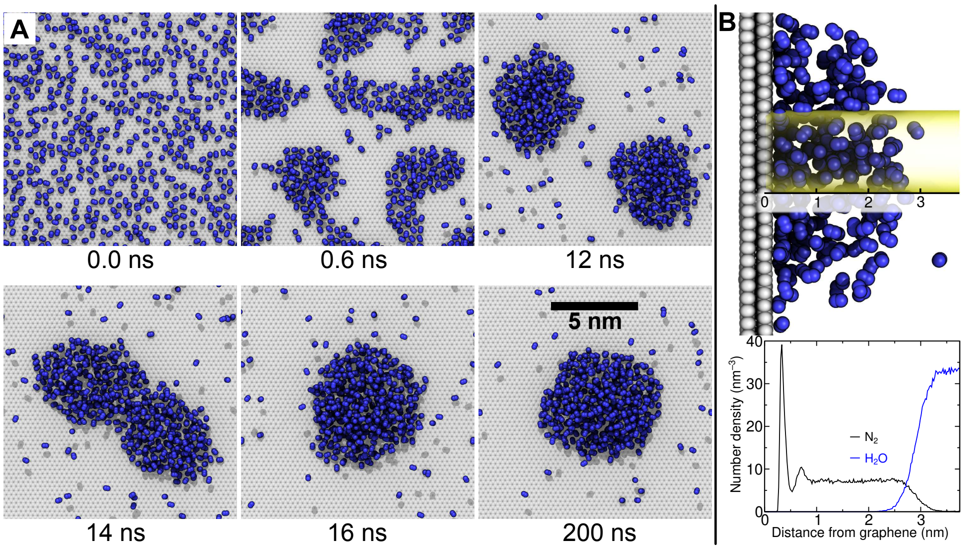

Figure S1: Spontaneous formation of dense bubble-like aggregates of N_2 at the graphene–water interface. **(A)** The simulation began with N_2 molecules distributed across the graphene surface at a density of 3.7 molecules/nm^2 and was evolved for 200 ns. Snapshots of the simulation are shown for several different times. Interatomic forces between N_2, water, and graphene were those of Peng et al.[1] (Peng-SPCE). For clarity, water molecules are not shown. **(B)** Number density of N_2 and water as a function of distance from the graphene surface for a region (translucent yellow cylinder) of the aggregate near its center. The density profile of these aggregates appears quite similar to that reported by Peng et al.[1] for their dense gas layers.

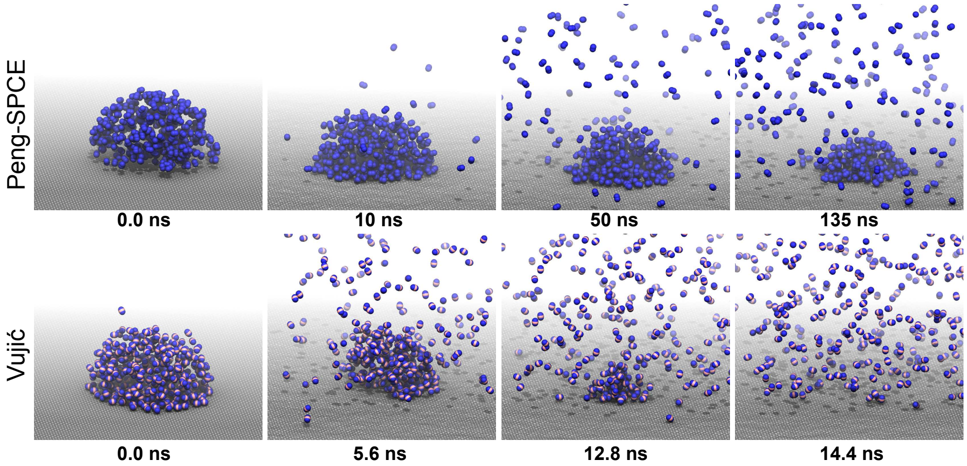

Figure S2: Evolution of hemispherical aggregates of N_2 at the graphene–water interface with supersaturated N_2 for two sets of force field parameters: the Peng-SPCE model[1] (top) and the Vujić model.[2]

Table S1: Free energy to form adsorbate–adsorbate pairs at the graphene–water interface and calculation of a wetting parameter ($\Delta A_{\mathrm{aq}\to\mathrm{ads}}/\Delta A_{\mathrm{pair}}$).

molecule	$\Delta A_{\mathrm{aq}\to\mathrm{ads}}$ (kcal/mol)	ΔA_{pair} (kcal/mol)	$\frac{\Delta A_{\mathrm{aq}\to\mathrm{ads}}}{\Delta A_{\mathrm{pair}}}$
N_2	−1.11	−1.02	1.1
hexane	−4.76	−2.47	1.9
octane	−6.12	−3.75	1.6
decane	−7.84	−5.06	1.5
dodecane	−9.29	−6.58	1.4
pentadecane	−11.64	−8.81	1.3
hexadecane	−12.10	−9.28	1.3
octadecane	−14.34	−10.74	1.3
2-methylheptane	−6.07	−3.12	1.9
isooctane	−4.87	−2.01	2.4
7-ethyltetradecane	−11.40	−4.59	2.5
ethanol	−2.22	−0.58	3.8
toluene	−5.07	−1.25	4.0
limonene	−6.87	−1.99	3.5
N_2 (no water)	−2.04	−0.18	11.4
octane (no water)	−11.80	−1.01	11.7

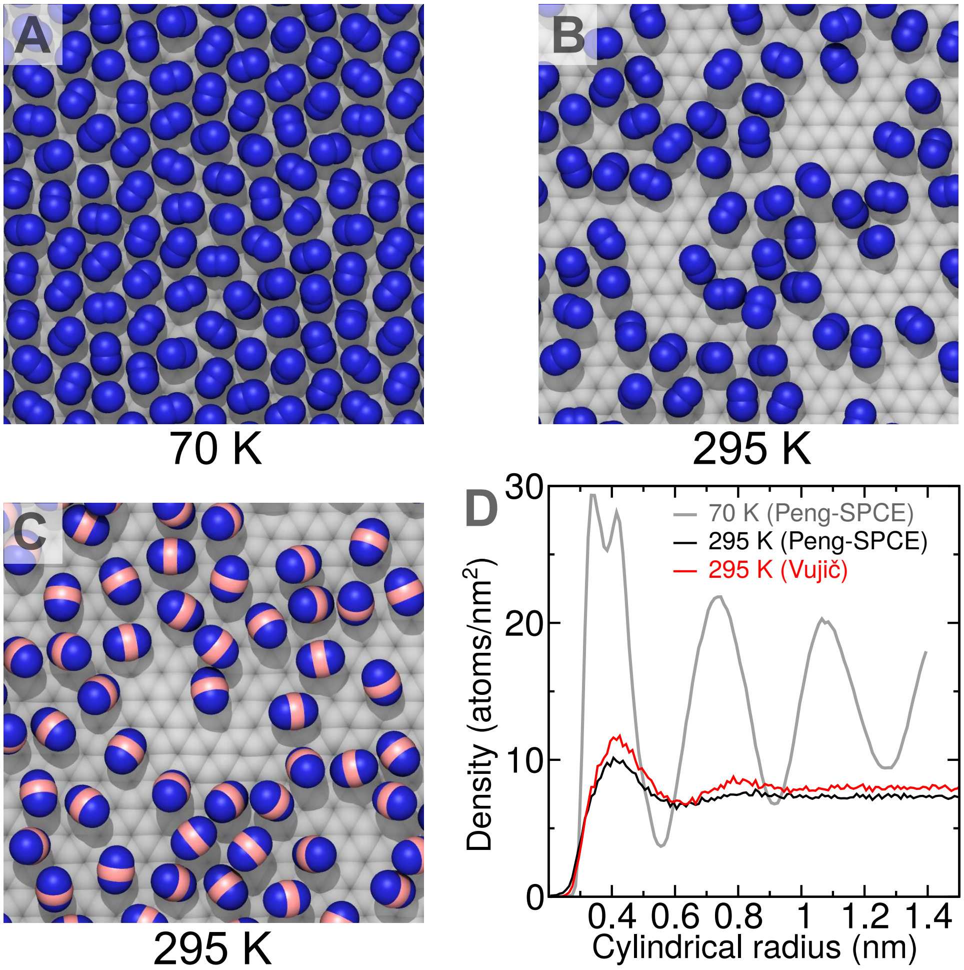

Figure S3: Configurations of N_2 molecules on graphene. **(A)** Ordered arrangment of N_2 molecules at an interface between liquid N_2 and graphene at 70 K in a simulation using the Peng-SPCE model. **(B–C)** Disordered arrangment of N_2 molecules at the graphene–water interface at room temperature in simulations using the Peng-SPCE (B) or Vujić (C) models. **(D)** Cylindrical distribution function for the first layer N_2 molecules on the graphene surface.

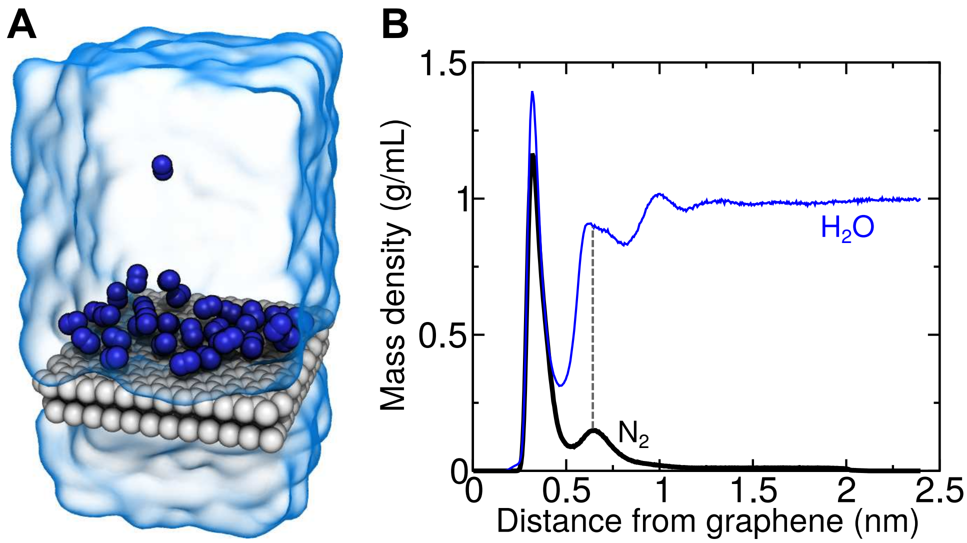

Figure S4: Density profile of N_2 at the graphene–water interface showing no sign of N_2 occupying the low density region between water layers. **(A)** Simulation snapshot of a thin N_2 layer atop a patch of graphene in water. **(B)** Density profile of N_2 and water along the axis perpendicular to the graphene surface.

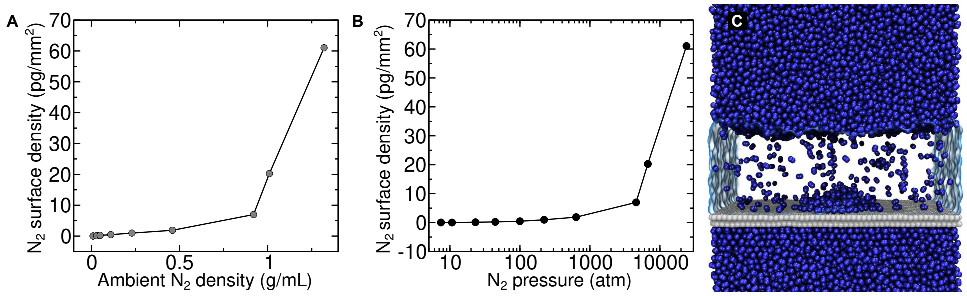

Figure S5: Simulations to determine the ambient N_2 concentration required to stabilize dense N_2 aggregates at the graphene–water interface. **(A)** Areal mass density of N_2 in the first solvation layer (less than 0.5 nm from the graphene sheet) as a function of the ambient volumetric mass density of the N_2 phase. The values are calculated after 120 ns of equilibration. The N_2 aggregate initially at the graphene–water interface dissipates before 120 ns in all simulations except for the one at the highest ambient N_2 density. It appears stable in the latter simulation. **(B)** Areal mass density of N_2 in the first solvation layer as a function of the system pressure. The pressure required to stabilize the aggregrate exceeds the pressure at which N_2 is fluid, so the validity of the highest pressure simulation is questionable. **(C)** Cross section of a simulation in which the N_2 aggregate appears stable. Nitrogen and graphene carbon atoms are shown, respectively, as blue and gray spheres. For clarity, explicit water molecules are not shown, but represented by a transparent cyan surface.

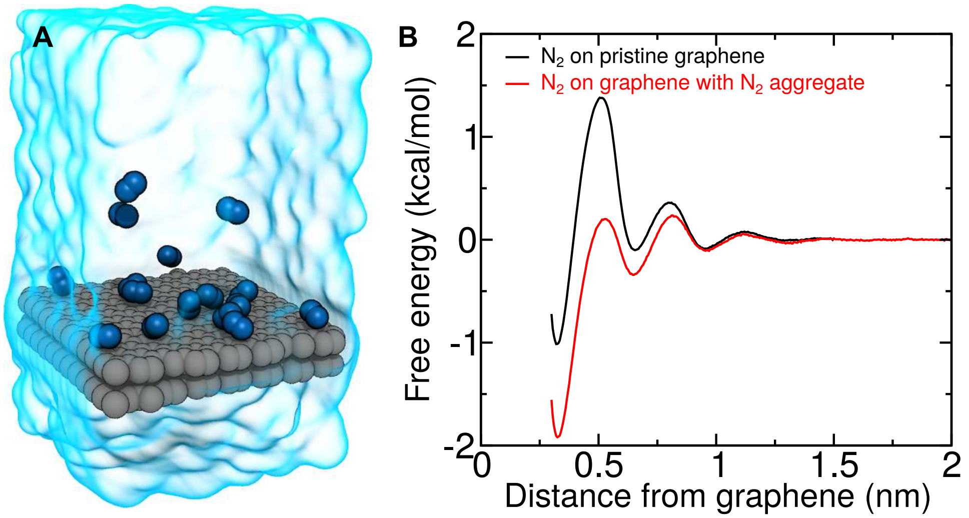

Figure S6: Cooperative effects in adsorption of the N_2 to the graphene–water interface. Binding an additional N_2 molecule becomes somewhat more favorable when the interface is already occupied by N_2. **(A)** Simulation system used for the calculation. **(B)** Potential of mean force on a single N_2 molecule as a function of distance from the upper layer of graphene for a pristine graphene–water interface or for one already occupied by an N_2 aggregate.

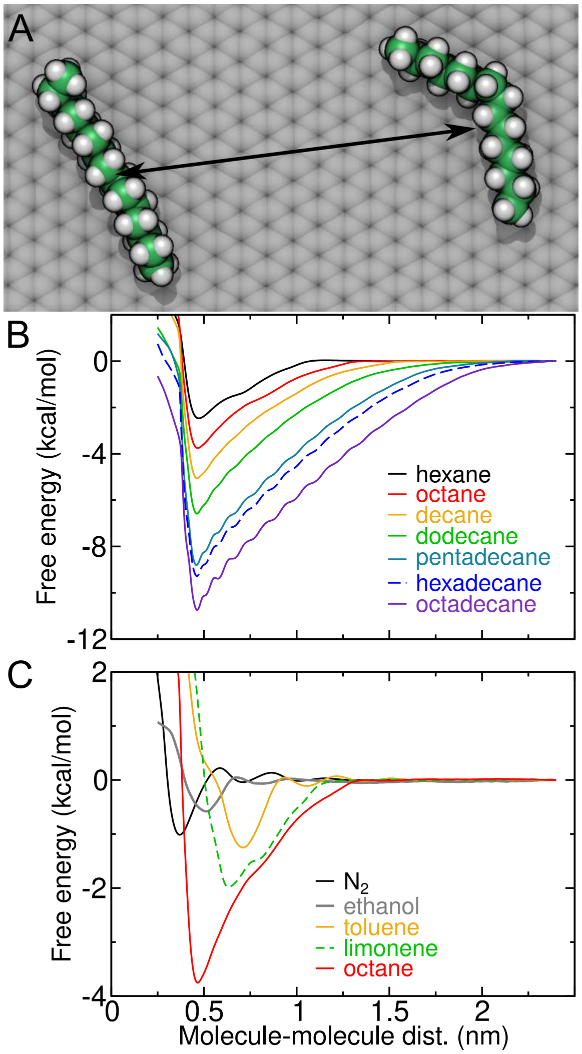

Figure S7: Free energy of pair formation at the graphene–water interface. **(A)** Image of two pentadecane molecules at the graphene–water interface. The transition coordinate for the free energy calculations is $\rho = \sqrt{(x_1 - x_2)^2 + (y_1 - y_2)^2}$ the distance between the centers of mass of the two molecules projected into the xy plane (the plane parallel to the graphene surface). **(B)** Free energy as a function of ρ for a series of alkanes. The geometric (Jacobian) contribution to the free energy has been removed. **(C)** Free energy as a function of ρ for other VOCs. Octane is included for reference.

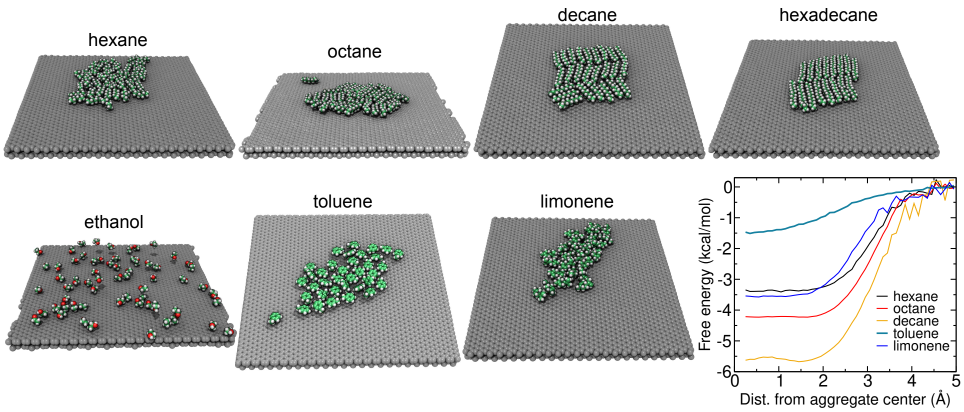

Figure S8: Configuration of aggregates of 42 hexane, 32 octane, 25 decane, 16 hexadecane, 85 ethanol, 36 toluene, and 25 limonene molecules at the graphene–water interface. (bottom-right) Free energy as a function of distance from the aggregate center calculated by $-k_\mathrm{B}T\ln(h(\rho)) + k_\mathrm{B}T\ln(2\pi\rho)$, where $h(\rho)$ is a histogram of the cylindrical radial coordinate ($\rho = \sqrt{x^2 + y^2}$) of the molecules relative to the center of mass of the aggregate and the second term removes geometric (Jacobian) contribution to the free energy.

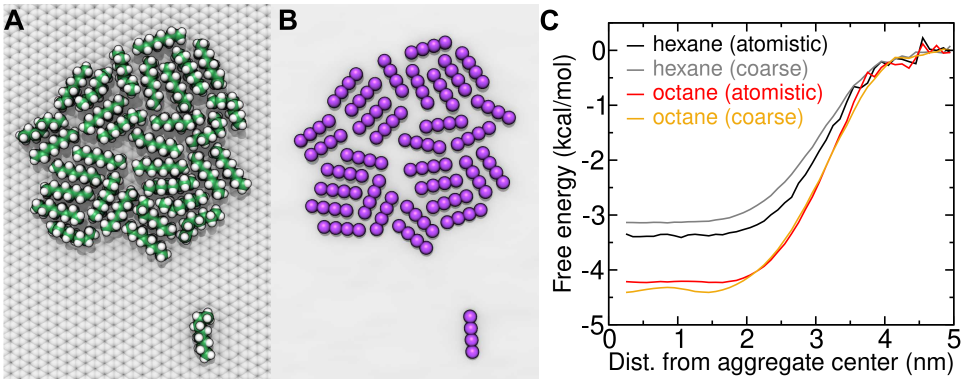

Figure S9: Coarse-grain model of straight-chain alkanes at the graphene–water interface. **(A)** All-atom representation of octane aggregate at the graphene–water interface. Explicit water molecules are not shown. **(B)** Coarse-grain model of the same octane aggregate. The coarse-grain model does not include explicit solvent, but is explicitly constructed to reproduce the free energy of transfer from the gas phase to isolated adsorbed phase ($\Delta A_{\mathrm{gas}\rightarrow\mathrm{aq}} + \Delta A_{\mathrm{aq}\rightarrow\mathrm{ads}}$) as calculated in the all-atom simulations. Each bead represents two carbon atoms. **(C)** Bead–bead interactions were calibrated so that the distribution of adsorbate molecules (hexane or octane) relative to the center of mass of the aggregate agreed decently between the all-atom and coarse-grain models. Fifty different bead–bead interaction parameters were tried and the best performing one ($\epsilon_{\mathrm{bead}} = 0.86$ kcal/mol and $\epsilon_{\mathrm{bead}} =3.795$ Å), whose results are graphed in this panel, was used for the GCMC calculations.

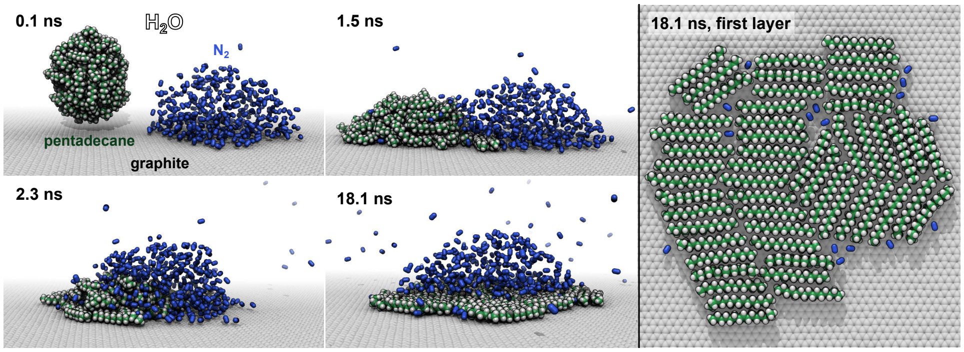

Figure S10: Simulation of a pentadecane droplet in water near an N_2 aggregate at the graphene–water interface under supersaturated conditions. The right panel is a cross section showing only the N_2 nearest the graphene surface. As might be expected from the favorable $\Delta A_{\mathrm{aq}\rightarrow\mathrm{ads}}$ of heavy alkanes compared to N_2, we found that they displace N_2 from the graphene–water interface. A droplet of pentadecane released near an N_2 aggregate eventually merges with it.

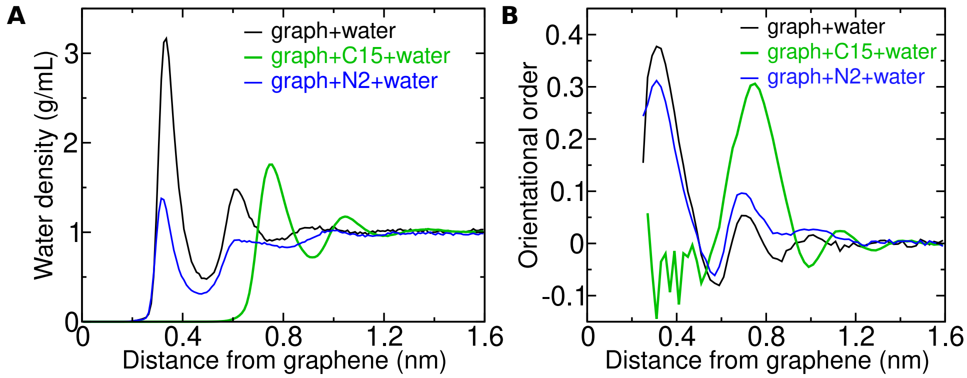

Figure S11: Water structure at graphene–water interfaces. **(A)** Mass density of water as a function of distance from the graphene sheet for a pristine graphene–water interface, a graphene–water interface occupied by a complete pentadecane monolayer, and a graphene–water interface with an N_2 aggregate (supersaturated solution of N_2). The density of water in the first solvation layer at the pristine graphene–water interface is more than triple that in bulk water. The density of water is also enhanced on top of the pentadecance monolayer, but the magnitude is considerably less. **(B)** Orientational order parameter $\frac{1}{2}(3\cos^2\theta - 1)$ of water molecules at these graphene–water interfaces. Here, θ is the angle between the z-axis (perpendicular to the graphene sheet) and the normal to the plane going through the centers of a water molecule's atoms. Near the graphene–water interface, the plane of each water molecule tends to align parallel to the plane of the graphene.

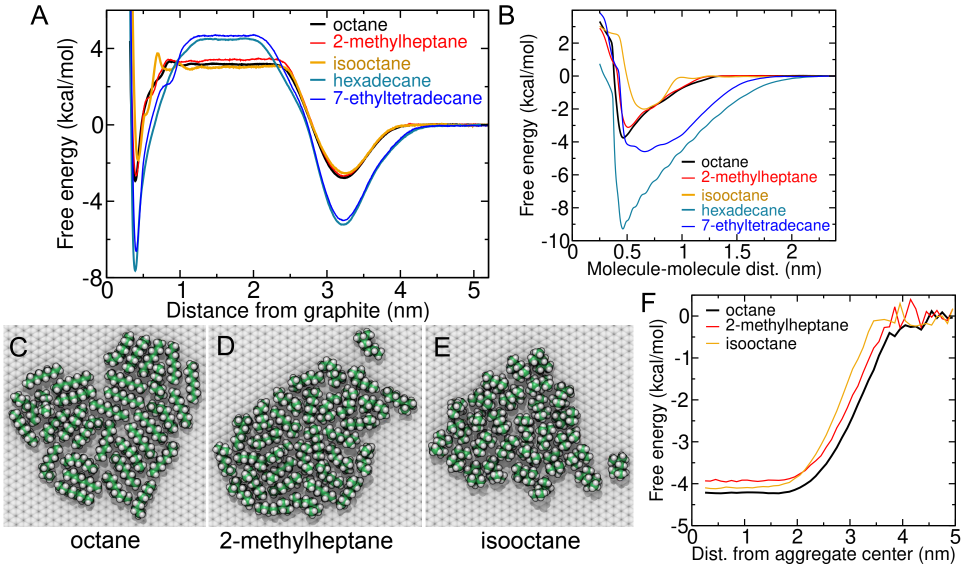

Figure S12: Comparison of adsorption thermodynamics of branched and straight-chain alkanes. Straight-chain octane is compared to 8-carbon isomers 2-methylheptane and isooctane (2,2,4-trimethylpentane). **(A)** Free energy for transfer from the gas phase, through the aqueous phase, to the graphene–water interface. Branched alkanes exhibit less favorable adsorption. **(B)** Free energy for forming pairs of adsorbed alkanes at the graphene–water interface. The free energy is calculated as a function of the cylindrical radial distance ($\rho = \sqrt{(x_1 - x_2)^2 + (y_1 - y_2)^2}$). The geometric (Jacobian) term ($-kT \ln(2\pi\rho)$) has been removed so that the free energy plateaus at large distances. The formation of pairs is considerably less favorable for branched isomers as compared to their straight-chain congeners. **(C–E)** Dense monolayer aggregates formed from octane isomers at the graphene–water interface. Greater amounts of branching lead to more disordered aggregates. **(F)** Free energy as a function of distance from the aggregate center calculated by $-k_{\mathrm{B}}T \ln[h(\rho)] + k_{\mathrm{B}}T \ln[2\pi\rho]$, where $h(\rho)$ is a histogram of cylindrical radial coordinate of the molecules relative to the center of mass of the aggregate and the second term removes geometric (Jacobian) contribution to the free energy. While the free energy for joining the aggregate is less favorable for the branched octane isomers than for n-octane, the difference is not as dramatic as for forming pairs.

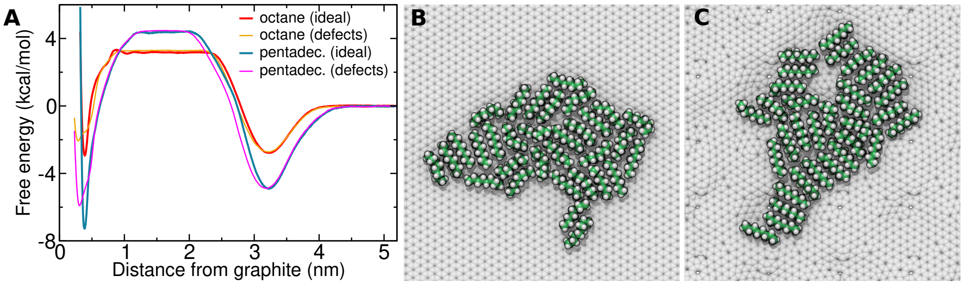

Figure S13: Effect of graphene defects on adsorption at the graphene–water interface. **(A)** Free energy for transfer from the gas phase to the aqueous phase to graphene–water interfaces with ideal structure and with a defect-rich structure (61:10:8:1 ratio of 6, 5, 7, and 8-membered carbon rings). The defects create undulations that broaden the free energy minima, but result in an overall reduction in the adsorption affinity. **(B)** Snapshot from a simulation of an octane aggregate at the graphene–water interface for ideal graphene. **(C)** Snapshot from a simulation of an octane aggregate at the graphene–water interface for a defect-rich graphene sheet. The octane molecules prefer concave regions and spend less time in convex regions.

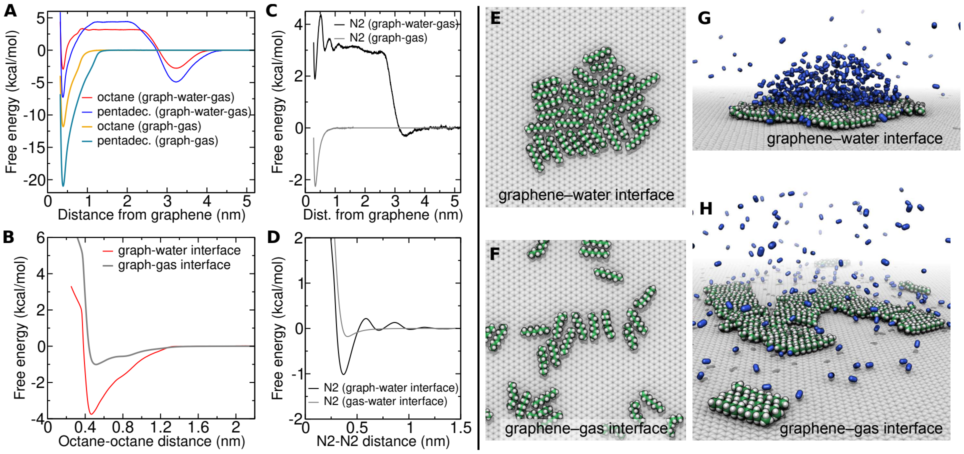

Figure S14: Comparison of adsorption at the graphene–water and graphene–gas interfaces. **(A)** Free energy for transfer from the gas phase to the graphene–water interface or the graphene–gas interface for alkanes. The latter is significantly more favorable. **(B)** Free energy for octane–octane pairs at the graphene–water and graphene–gas interfaces. Pairing is much less favorable at the graphene–gas interface than at the graphene–water interface. **(C)** Free energy for transfer from the gas phase to the graphene–water interface or the graphene–gas interface for N_2. **(D)** Free energy for N_2–N_2 pairs at the graphene–water and graphene–gas interfaces. **(E–F)** Comparison of octane aggregates at the graphene–water and graphene–gas interfaces. The cohesion of the aggregates is much greater in the presence of water than in its absence. **(G)** N_2 aggregate atop a pentadecane monolayer at the graphene–water interface. **(H)** Same system as panel G except in the absence of water. The pentadecane aggregate is much less compact and no aggregation of N_2 is apparent.

References

1. H. Peng, G. R. Birkett and A. V. Nguyen, *Langmuir*, 2013, **29**, 15266–15274.
2. B. Vujić and A. P. Lyubartsev, *Model. Simul. Mater. Sc.*, 2016, **24**, 045002.
